# Sensorimotor Model of Obstacle Avoidance in Echolocating Bats

**DOI:** 10.1371/journal.pcbi.1004484

**Published:** 2015-10-26

**Authors:** Dieter Vanderelst, Marc W. Holderied, Herbert Peremans

**Affiliations:** 1 Active Perception Lab, University of Antwerp, Antwerp, Belgium; 2 School of Biological Sciences, University of Bristol, Bristol, United Kingdom; ISRAEL

## Abstract

Bat echolocation is an ability consisting of many subtasks such as navigation, prey detection and object recognition. Understanding the echolocation capabilities of bats comes down to isolating the minimal set of acoustic cues needed to complete each task. For some tasks, the minimal cues have already been identified. However, while a number of possible cues have been suggested, little is known about the minimal cues supporting obstacle avoidance in echolocating bats. In this paper, we propose that the Interaural Intensity Difference (IID) and travel time of the first millisecond of the echo train are sufficient cues for obstacle avoidance. We describe a simple control algorithm based on the use of these cues in combination with alternating ear positions modeled after the constant frequency bat *Rhinolophus rouxii*. Using spatial simulations (2D and 3D), we show that simple phonotaxis can steer a bat clear from obstacles without performing a reconstruction of the 3D layout of the scene. As such, this paper presents the first computationally explicit explanation for obstacle avoidance validated in complex simulated environments. Based on additional simulations modelling the FM bat *Phyllostomus discolor*, we conjecture that the proposed cues can be exploited by constant frequency (CF) bats and frequency modulated (FM) bats alike. We hypothesize that using a low level yet robust cue for obstacle avoidance allows bats to comply with the hard real-time constraints of this basic behaviour.

## Author Summary

Echolocating bats can fly through complex environments in complete darkness. Swift and apparently effortless obstacle avoidance is the most fundamental function supported by biosonar. Despite this, we still do not know which acoustic cues, from among the many possible cues, bats actually exploit while avoiding obstacles. In this paper, we show using spatial simulations (2D and 3D) that the Interaural Intensity Difference (IID) and travel time of the first millisecond of the echo train in combination with alternating ear positions provide robust and reliable cues for obstacle avoidance. Simulating the echoes received by a flying bat, we show that simple phonotaxis can steer a bat clear from obstacles without performing 3D reconstruction of the layout of the scene. As such, this paper presents the first computationally explicit explanation for obstacle avoidance in realistic and complex 3D environments. We hypothesize that using low level yet robust cues for obstacle avoidance allows bats to comply with the hard real-time constraints of this basic behaviour.

## Introduction

Rhinolophidae are echolocating bats specialized in hunting for airborne prey among vegetation using echolocation. To cope with clutter echoes returning from vegetation they employ a unique sensorial strategy for detecting prey. They emit long narrow-band pulses and listen for frequency and amplitude shifts, so called glints, in the echoes caused by fluttering prey [1]. Echoes from stationary obstacles do not contain these glints and do not interfere with the detection and localization of prey [2].

While the sensorial adaptations of Rhinolophidae for prey detection have been extensively researched (see [1] for a review), the cues supporting the ability of these bats to navigate and orient in cluttered environments have received much less attention. Nevertheless, their ability to navigate small spaces [3–6] and their well-studied echolocation apparatus [1, 7] makes them an interesting taxon to study how echolocating bats avoid obstacles in natural environments. Indeed, as argued in the discussion, understanding the cues Rhinolophidae use to negotiate space is potentially informative about how other bats using frequency modulated pulses could avoid obstacles as well.

It would seem that Rhinolophidae, using long narrowband signals, lack both the bandwidth and the temporal resolution available to bats using short broadband signals. Indeed, bats using broadband signals typically shorten their calls (typically 1–3 ms [8]) and increase the bandwidth when moving into cluttered spaces [8]. Rhinolophidae, in contrast, negotiate cluttered space using much longer (about 10–50 ms) and narrowband signals that seem not particularly well suited for obstacle avoidance. Indeed, while Rhinolophidae also shorten their calls and increase the bandwidth when moving into cluttered space [9, 10], their calls remain longer and more bandwidth limited than those of FM bats under the same conditions.

The characteristic cyclical pinna movements shown by Rhinolophidae [11, 12] have been suggested to compensate for the lack of spatial cues available to bats relying on broadband calls. Mogdans et al. [3] performed behavioural experiments to test specifically the role of these ear movements for obstacle avoidance based on Interaural Intensity Differences (IIDs). The hypothesis [3, 10] that the moving ears generate changing IIDs encoding the reflector position in both the horizontal and the vertical plane was found by these authors to be in agreement with the results from their wire-avoidance experiments and put forward as a possible explanation for the bats’ obstacle avoidance ability. Since then, simulation studies and robotic experiments have corroborated that these ear movements do indeed provide various localization cues that would allow localizing individual reflectors, such as prey items [13–15]. However, natural environments encountered by bats are typically made up of objects that consist of many stochastic reflectors returning many overlapping echoes [16]. Therefore, for 3D localization of reflectors, e.g. based on typical ear movement induced IID patterns, to be considered a plausible mechanism underlying the obstacle avoidance abilities of bats, it has to be proven first that such a localization capability is robust in the presence of multiple overlapping echoes. Hence, while it has been shown that pinnae movements play a significant role in obstacle avoidance [3], it is still not clear what information Rhinolophidae extract from such pinna movements to allow them to avoid natural (and complex) obstacles.

To complement behavioural experiments, we use the synthetic methodology, i.e. understanding natural systems by building artefacts [17–19], computer simulations, in this case, to study bat obstacle avoidance behaviour. In particular, we propose a sensorimotor system that does not rely on the bat reconstructing the 3D spatial layout of reflectors from the echoes, but instead relies on the dynamics of the bat-obstacle interaction to result in obstacle avoidance behaviour. A similar approach is taken in ref. [20] for prey-catching behaviour in echolocating bats assuming that only a single reflecting target is present giving rise to a unique isolated echo. This assumption is warranted in the case of prey-catching behaviour as the bat can choose to hunt away from clutter [8] or take active measures to separate the echoes from the foreground prey item from the clutter background ones (e.g. [21, 22]). In contrast, realistic obstacles, e.g. foliage and/or man-made structures, will always give rise to multiple overlapping echoes [16].

The sensorimotor system we propose is intentionally kept as simple as possible. It uses IID and time delay of the first echo onset in combination with alternating pinna movements to guide the bat. In particular, it processes only the first millisecond of the echo train. Furthermore, it does not need the right and left ear echo signals to be segmented into contributions from individual reflectors, as would be required by any approach that reconstructs the spatial layout of the bat’s surroundings. While approaches that attempt to reconstruct the spatial layout of the environment first as a prerequisite for obstacle avoidance [23, 24], when successful, are clearly sufficient to explain such behaviour, we aim to show with the proposed sensorimotor system that such a reconstruction capability is not a necessary condition. The main advantage of the proposed obstacle avoidance mechanism is that because of its simplicity as well as its reliance on the first millisecond of the echo train only it can react very rapidly to the relevant information contained in an otherwise very complex echo signal consisting of many overlapping echoes. This allows the system to respond appropriately under hard real-time conditions independent of the complexity of the environment.

In this paper, we first present the environments used to simulate the echoes received by a bat moving through realistic, cluttered spaces. Next, we propose a sensorimotor system that results in obstacle avoidance behaviour by extracting echo delay and IID information from the onset of the first echo in combination with alternating pinna movements. Finally, we test the performance of the sensorimotor system in simulated 2D and 3D environments showing that despite its simplicity the system can avoid obstacles in a complex environment without the need to reconstruct the 3D spatial layout of the reflectors present.

## Methods

### Environments

We tested the proposed sensorimotor system both in environments that were artificially generated and in environments derived from 3D laser scans of real bat habitats. Below we discuss the construction of both types of test environments.

#### Artificial environments

The sensorimotor system was tested in both 2D and 3D artificial environments. We first test the proposed controller in 2D environments in addition to using the more realistic 3D environments for the following reasons.

Since originally pioneered by early bat researchers (See [25, 26] for early references) many obstacle avoidance tests with bats have been conducted by flying them through an array of vertical or horizontal wires, e.g. [3, 25, 27, 28]. The number of wires touched by the bats is taken as a measure of their obstacle avoidance capacity. These experiments essentially test the obstacle avoidance of bats in two dimensions allowing one to assess obstacle avoidance separately along the horizontal and the vertical dimension. For example, Mogdans et al. [3] flew *R. ferrumequinum* bats through a row of wires spaced 15 cm apart before and after obstructing their typical ear movements. These authors found that incapacitating the ear movements resulted in an increase in the number of horizontal wires touched by the bats. At the same time, avoiding the vertical wires was not influenced by fixating the ears. Including two-dimensional artificial environments allows us to match the experimental conditions of Mogdans et al. [3].

Furthermore, the behaviour of the sensorimotor system in 2D environments is easier to visualize and analyze than the 3D case. Hence, 2D simulations allow us to demonstrate the behaviour of the controller more clearly.

Finally, horizontal 2D environments are relevant as many real environments encountered by bats give rise to essentially 2D obstacle avoidance problems, i.e. avoidance manoeuvres can be executed in a plane. When following flight corridors or navigating amongst trees, bats can usually maintain a fixed altitude while avoiding obstacles. Therefore, the horizontal analysis represents a situation commonly faced by bats.

One limitation of the 2D simulations is that all reflectors were positioned in the flight plane. In reality, even if the bat moves in a plane, the echoes from above and below the flight plane would also interfere with processing of the echoes from the flight plane. These echoes were not modeled in the 2D environments. However, they were modeled in all, including the laser scanned, 3D environments discussed below.

##### Regularly spaced artificial environments

Mogdans et al. [3] tested the obstacle avoidance capacity of *R. ferrumequinum* by flying it through a single row of horizontal or vertical wires spaced 15 apart. To mimic these experimental settings we generated a 2D array of regularly spaced point reflectors on a disk with a radius of 5 m (See Fig 4 for examples). The reflectors were arranged on a hexagonal grid and spaced 15 cm apart. The target strength for the point reflectors was set to −66 dB which corresponds to a wire with a diameter of 0.16 mm as reported in ref. [29]. We generated both a vertical and horizontal version of the regularly spaced obstacles testing the ability of the controller to avoid horizontal and vertical wires respectively.

In reality, *R. rouxii* would not be capable of sustained flight in environments with such dense wire distribution as the bat would be required to keep its wings folded to pass the wires. For this reason, the obstacle area is usually limited to one or a few rows of wires in real behavioural experiments. However, by disregarding the unrealistic aerodynamic demands, the dense array of wires simulated here allows us to effectively evaluate the performance of the proposed obstacle avoidance strategy in dealing with the wire spacings used in the behavioural experiments of Mogdans et al. [3].

##### Heterogeneous artificial environments

The heterogeneous cluttered environment in which Rhinolophidae typically operate was modeled as a large number of point reflectors placed in the volume of either a sphere (3D, see Fig 6) or on the surface of a disk (2D, see Fig 5). Filling the sphere or disk with reflectors was done in two stages. First, a number of centre points were chosen at random (uniform distribution). Next, for each centre point a cluster of reflectors was generated by drawing locations from a 3D or 2D normal distribution of which the covariance matrix was randomized [30]. This two stage process resulted in point reflectors that were clustered in space (See Figs 5 and 6 for examples). The generation of the environments was controlled by a number of parameters listed in Table 1.

**Table 1:** Parameters used to generate heterogeneous artificial environments mimicking the cluttered habitats of Rhinolophidae.

Parameter	Value
2D simulations	
Radius of simulation environment	20 m
Number of reflector clusters	100
Number of reflectors per cluster	250
Variance of X,Y position of reflectors	0.5 m
3D simulations	
Radius of simulation environment	20 m
Number of reflector clusters	500
Number of reflectors per cluster	500
Variance of X,Y & Z position of reflectors	1.5 m

##### Tilted torus environment

A final artificial environment in which the controller was tested consisted of a torus lined with reflectors. The major diameter of the torus was 10 meter. The minor diameter was 2 meter. The torus was tilted by 45 degrees (See Fig 7c). This forces the controller to control both elevation and azimuth in order to trace the torus. Hence, this environment tests the controller for its ability to follow a corridor in both elevation and azimuth.

The torus was lined with reflectors spaced approximately 10 cm apart. The target strengths of the reflectors *s*_*i*_ were set to vary randomly in the interval −46 to −34 dB. This corresponds to −40 dB, the approximate target strength of a sphere with diameter 5 cm [31], plus and minus 6 dB.

##### Laser scanned environments

In addition to the regularly spaced and heterogeneous artificial environments, we also tested the sensorimotor system using arrays of point reflectors that were derived from 3D laser scans of two real bat habitats.

Two bat habitats, a patch of fir forest and a commuting corridor in a forest, were scanned using the Panorama Laser scanner IMAGER 5003 (Zoller + Fröhlich). This system has a maximum range of 53 m and a 360 degrees field of view. The scanner was operated in High-Resolution Modus resulting in 125 cm^3^ voxels (i.e. voxels of 5 × 5 × 5 cm). To obtain sufficient data to reconstruct a complete model of both environments, individual scans from different positions were combined, depending on the density of trees and occlusions from the flight corridors of bats [32]. The scanned volume for the fir forest measured 20 × 20 m and was 12 m high (39 million 125 cm^3^ voxels). The flight corridor was 20 m long and about 2 m wide. The corridor was flanked by rows of vegetation of about 5 meters wide. The volume contained 80 million 125 cm^3^ voxels). Due to computer memory limitations, data for both environments was smoothed and subsampled at a resolution of 3375 cm^3^ (i.e. voxels of 15 × 15 × 15 cm). Next the x, y and z-coordinates of occupied voxels were extracted and used as a collection of point reflectors for testing the algorithm in the same fashion as for the artificially generated point clouds.

Importantly, when scanning habitats using a Laser scanner, only outer surfaces of structures such as trees result in filled voxels. Moreover, only surfaces oriented towards the scanner can be detected. The inside and backside of structures are not visible to the scanner. Therefore, all voxels used here can be exposed to sound.

It should be noted that sparsely placed point reflectors are only approximate representations of large geometrical bodies such as tree trunks. These are expected to differ in target strength, the proportion of geometric attenuation (because reflected wave is non-spherical [33]) and the number of reflections that contribute to the first 1 ms of the echo train. Nevertheless, Yovel et al. [34] demonstrated that the power spectra of different types of plants could be well fitted by point clouds with mean spacings ranging from 16 to 20 cm (Table 1 in ref. [34]). Hence, while sparsely placed point reflectors are not realistic acoustic representations of large geometrical bodies, evidence suggests they are at least representative of leafy vegetation

### Calculation of echo strength

The intensity of the echo returning from each point reflector *i* was calculated for each call. The intensity *g*_*i*_ (in dB) of the echo received from reflector *i* is given by the sonar equation [31],

$$\begin{array}{c} g_{i}=g_{bat}+40\cdot {\text{log}}_{10}\frac{0.1}{r_{i}}+2\cdot \left(r_{i}-0.1\right)\cdot a_{f}+d_{\phi_{i},p}+s_{i}+c_{\phi_{i}} \end{array}$$ (1)

In Eq (1), *g*_*bat*_ is the intensity of the call at 10 cm from the mouth, in this paper taken to be 120 dB_*spl*_ [9]. The parameters *r*_*i*_, *a*_*f*_, *d*_*ϕ*_*i*_,*p*_, *s*_*i*_ give the range to reflector *i*, the atmospheric absorption at frequency *f* [35], the directional sensitivity *d*_*ϕ*_*i*_,*p*_ of the sonar apparatus of the bat for angle *ϕ*_*i*_ and pinnae position *p* (see below), and the echo strength *s*_*i*_ of the reflector respectively. Simon et al. [36] ensonified leaves for a range of aspect angles and found reflector strength to vary from −30 dB to −6 dB. Therefore, variations in aspect dependent reflector strength *s*_*i*_ were modelled by choosing the reflector strength randomly from a uniform distribution over this interval for each call.

As stated above, for the regularly spaced artificial environments mimicking the wire avoidance tests of Mogdans et al, [3] the reflector strength *s*_*i*_ was fixed at −66 dB corresponding to the target strength of a wire with a diameter of 0.16 mm [29]. In the torus environment, the reflector strength was chosen randomly from the interval −46 to −34 dB. This corresponds to −40 dB, the approximate target strength of a sphere with diameter 5 cm [31], plus and minus 6 dB.

In Eq (1), *c*_*ϕ*_*i*__ denotes an additional attenuation reflecting changes in cochlear sensitivity for different frequencies. The cochlea of Rhinolophidae is highly tuned to the species-specific constant frequency component of the call (Reviewed in [1]). While flying, these bats compensate the Doppler shift of the returning echoes by lowering the emission frequency. In doing this, they effectively ensure that echoes return with a frequency very close to the frequency their cochlea is tuned to, i.e. the reference frequency. However, the Doppler shift Δ*f*_*ϕ*_*i*__ of an echo depends on the heading direction *ϕ*_*i*_ of reflector *i* as follows,

$$\begin{array}{c} \Delta f_{\phi_{i}}=f_{emission}\cdot \frac{2\cdot v_{bat}}{v_{sound}}\cdot \text{cos}\;\phi_{i} \end{array}$$ (2)

**Fig 1:**
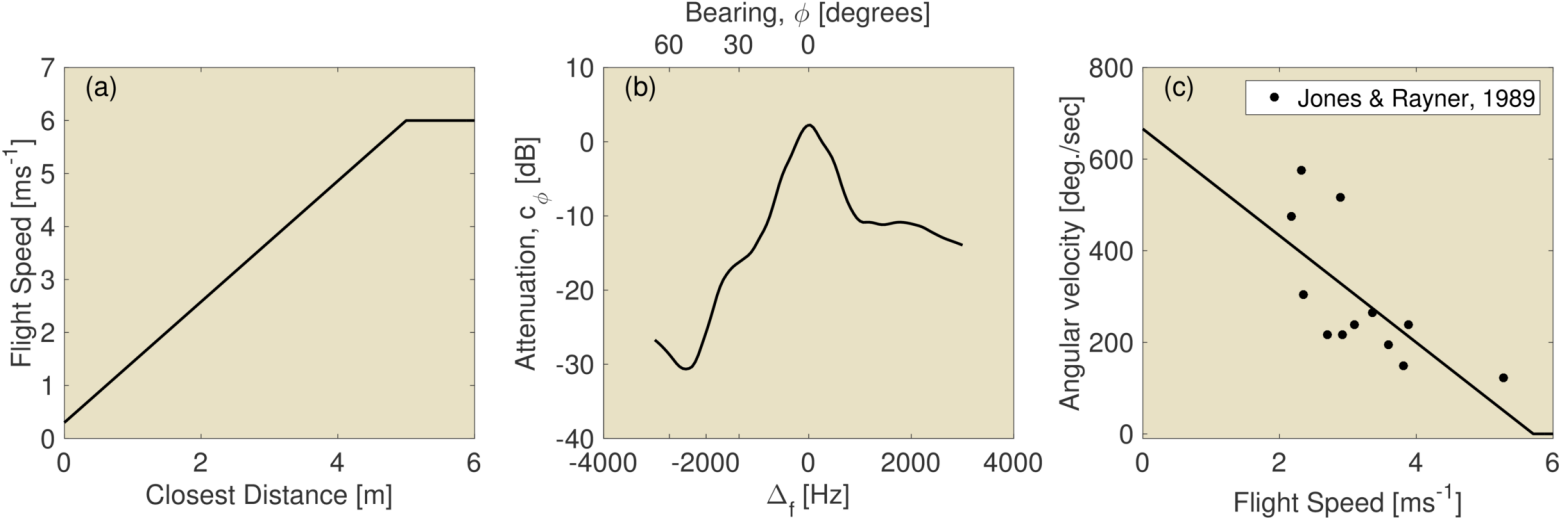
(a) The dependence of modeled flight speed on the distance to the closest obstacle. See text for a motivation of this curve. (b) Attenuation as a function of reflector bearing. Here depicted assuming *V*_*bat*_ = 6 ms^−1^. Neuweiler [7] reports on the change in detection threshold as a function of the difference between the echo frequency and the reference frequency for three individual *R. ferrumequinum*. The curve depicted in this figure was derived by averaging across the three individuals and was used to model the effect of varying Doppler shifts on the gain of the echo, i.e. *c*_*ϕ*_*i*__ in Eq (1). (c) Dependence of the modeled angular velocity on speed. See text for a motivation of this curve. The data plotted was taken from Jones and Rayner [6].

We were unable to find flight speed data for *R. rouxii*. However, bats weighing about 10 grams were reported to commute with a speed of 6 ms^−1^ [37, 38]. Therefore, we modelled the maximum speed of *R. rouxii* as *v*_*bat*_ = 6 *ms* ^−1^. *R. ferrumequinum* is capable of drastically reducing its flight speed when near an obstacle. Aldridge [4] reports a flight speed of about 0.3 ms^−1^ at the maximum turning rate for *R. ferrumequinum*. Moreover, this bat starts reducing its speed from about 5 meters before landing [9]. Hence, we model the flight speed of *R. rouxii* as 0.3 ms^−1^ and 6 ms^−1^ at 0 and 5 meter (and more) from the nearest obstacle respectively (See Fig 1a). We interpolate linearly between these points. Notice that this implies that the simulated flight speed in the regularly spaced artificial environments (see below) where obstacles are spaced 15 cm apart is maximally about 0.47 ms^−1^.

The details of how Rhinolophidae lower their emission frequency when faced with multiple reflectors with different Doppler shifts remain unknown. Experiments using masking tones [39] suggest the bats lower their emission frequency such that the frequency of the maximally Doppler shifted echo is close to the reference frequency (i.e. the frequency they are maximally sensitive to). However, the compensation exhibited depends also on the intensity and delay of the echoes as well as the time constant of the feedback loop [39, 40]. As a first order approximation, we assumed that the synthetic bat lowers its emission frequency by about 2.6 kHz to compensate the Doppler shift for reflectors with heading *ϕ* = 0 (at *v*_*bat*_ = 6 m/s and *f*_*emission*_ = 75 kHz). Lower flight speeds result in reduced Doppler shifts. This implies that we assume that reflectors *i* with *ϕ*_*i*_ > 0 return echoes with frequencies between 0 and about 2600 Hz below the reference frequency. Hence, in our simulations, we attenuate echoes for *ϕ*_*i*_ > 0 as bats are less sensitive to frequencies below the preferred frequency. The attenuation *c*_*ϕ*_*i*__ for each echo as a function of the heading angle *ϕ*_*i*_ was determined based on data reported by Neuweiler [7] (See Fig 1b). It should be noted that this simple implementation of the Doppler compensation mechanism overestimates the loss in sensitivity due to Doppler shifts. Indeed, we assume the maximum Doppler shift experienced (and, hence the decrease in emission frequency) is always equal to the hypothetical Doppler shift for an object with heading zero degrees—even if these echoes have large delays or low amplitudes. In reality, bats lower their frequency to a lesser extent when echoes have low intensity and/or long delays [39, 40].

In the current simulations, we modeled the bat *Rhinolophus rouxii* which uses constant frequency calls in the range 73–79 kHz [5]. We choose to approximate the call frequency using 75 kHz. The atmospheric absorption *a*_*f*_ at 75 kHz was set to 2.4 dB/m [35]. The directional sensitivity *d*_*ϕ*_*i*_,*p*_ of the synthetic bat’s hearing and emission for 75 kHz was taken from previous simulation studies [13, 14, 41]. The maximum gain of the head related transfer function was set to 4.5 dB at 75 kHz [42].

As pointed out above, experimental results confirm that the typical ear movements of Rhinolophidae support obstacle avoidance [3]. The continuous movement of the pinnae is approximated by modeling the directional sensitivity of the two extreme positions *p* of the ears. This is warranted by the fact that the controller proposed in this paper (detailed in the next section) only processes the onset of the echoes, i.e. the first millisecond. The available evidence [11, 12, 43] suggest that the pinnae are in the most extreme position at the onset of the echo and sweep to the inverse orientation while receiving the echo(es).

**Fig 2:**
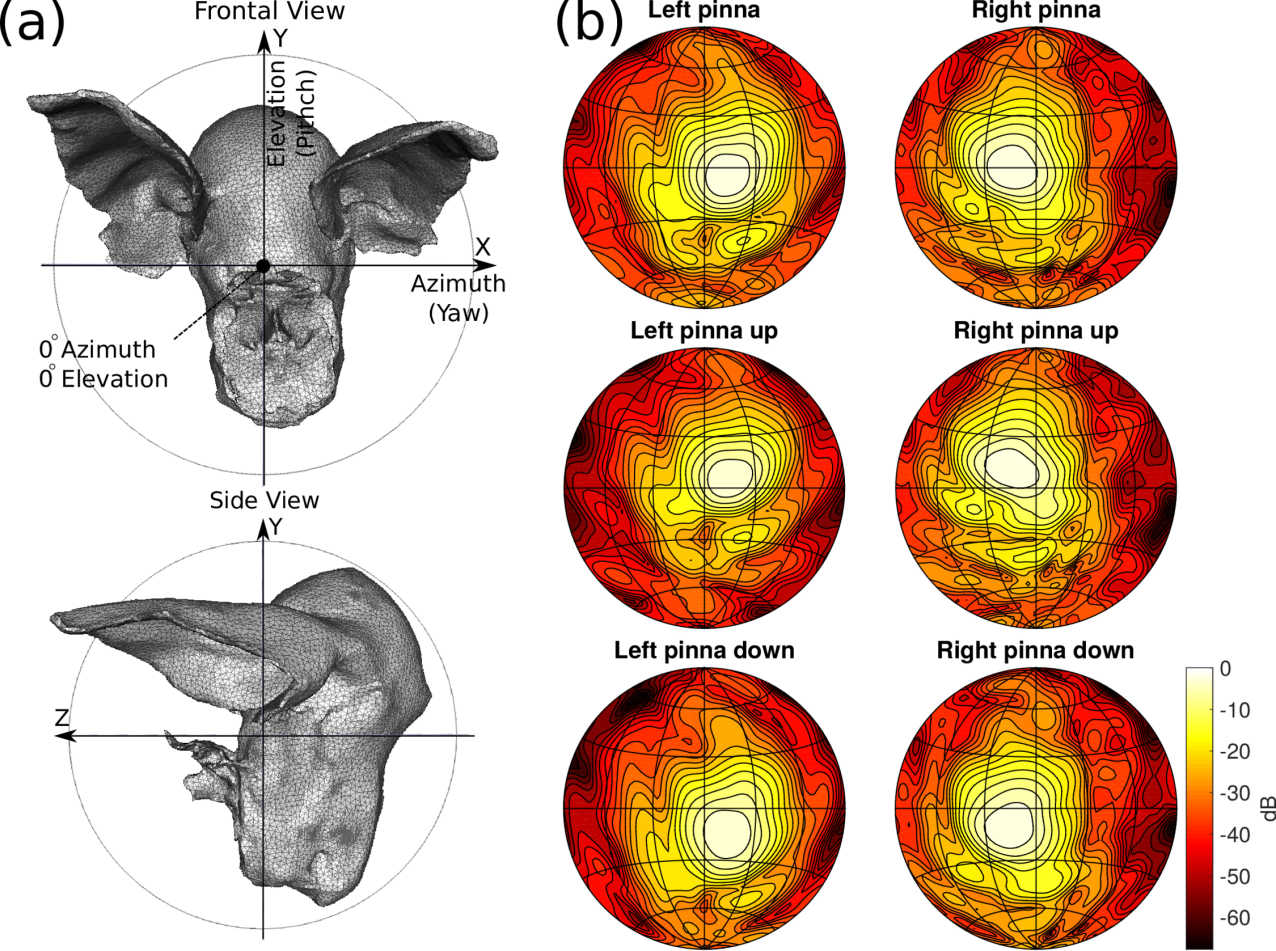
(a) Renderings of the 3D model used to simulate the directional sensitivity of the sonar system of *R. rouxii*. (b) The simulated directional sensitivity (combination of the head related transfer function (HRTF) and the emission beam directionality). Top row: the directional sensitivity of the model as depicted in (a). Middle row: directional sensitivity for the HRTF rotated 15 degrees upwards. Bottom row: Directional sensitivity for the HRTF rotated 15 degrees downwards. Note that the emission beam was not rotated (i.e. the HRTF was rotated with respect to the emission beam). The simulated directionality of the hearing and emission was taken from references [13, 14].

Pinna movements are simulated by rigidly rotating the hearing spatial sensitivity pattern before combining it with the emission directivity to obtain the complete directional sensitivity (see [13, 14] for details). Measurements have shown that the ears of Rhinolophidae do not undergo rigid rotations but instead deform while rotating [44]. However, current evidence leaves open the question whether the effects of this deformation on the hearing spatial sensitivity pattern is functionally relevant or not. Ref. [45] discusses the validity of modeling the ear movements as rigid rotations. The modeled head related transfer functions for the two pinna positions *p* are depicted in Fig 2.

### Derivation of the controller

**Fig 3:**
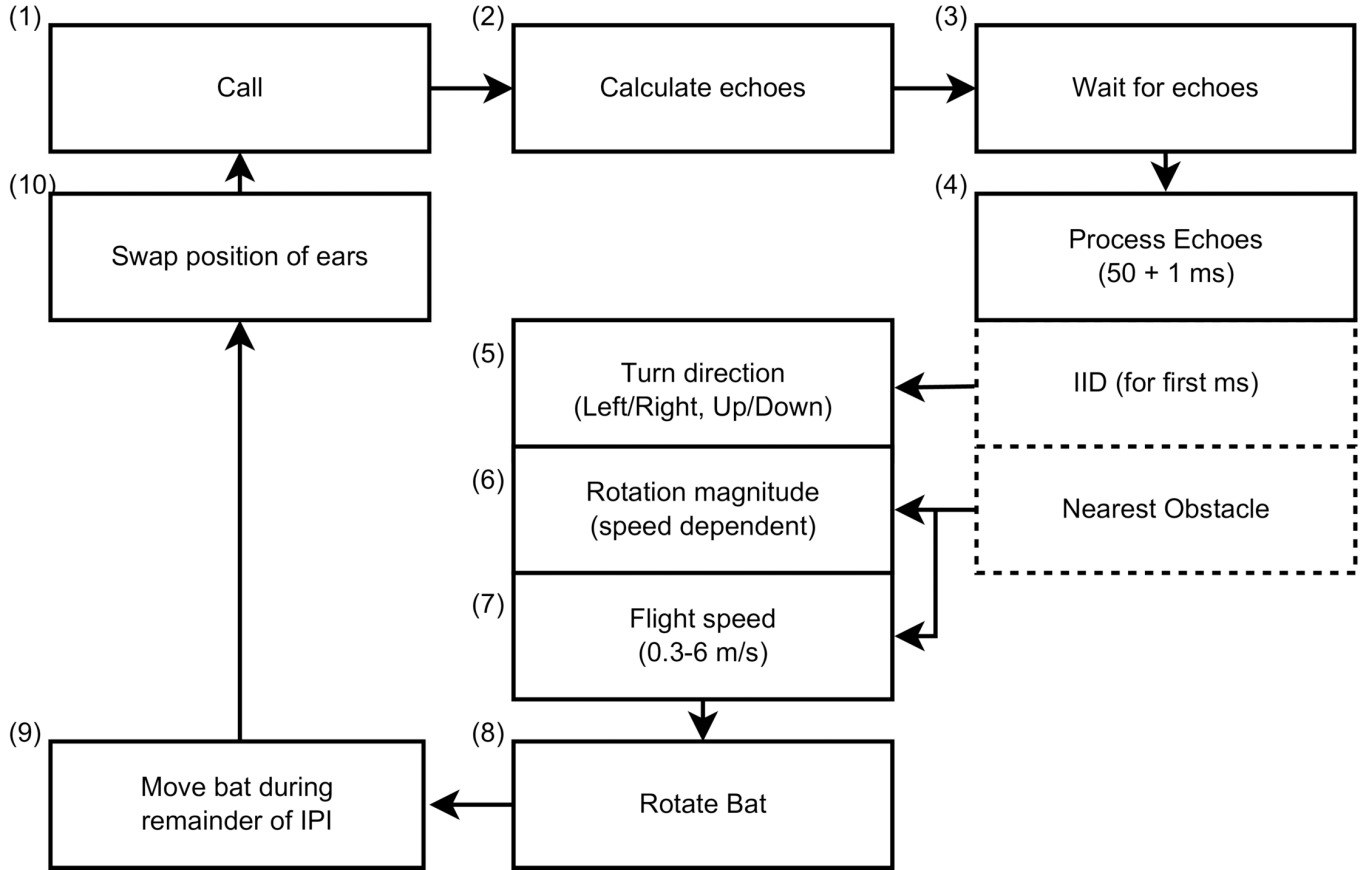
This diagram describes the simulations and the controller investigated in this paper. (1) The simulation starts with the bat emitting a call. (2) Next, the echoes returning from all point reflectors are calculated. (3) While waiting for the first echo to arrive, the controller keeps the current flight direction. (4) Moreover, 50 ms is allowed to process the onset of the echo train (1 ms). Based on the IID the rotation direction (5) is determined. The magnitude (6) depends on the flight speed. The new flight speed itself is chosen based on the distance to the nearest obstacle (7). The controller applies the determined rotation angle and (8) moves in the new direction for the remainder of the interpulse interval (9). Finally, the controller swaps the position of the ears before emitting the next call (10).

At the heart of the sensorimotor system responsible for obstacle avoidance behaviour we propose a biologically feasible controller that does not rely on explicit reconstruction of the 3D layout of individual reflectors to explore the possibility that Rhinolophidae can avoid obstacles without making use of a 3D model of the world. The controller is illustrated in Fig 3. We assume that the flight parameters are updated after every call based only on the echoes of the last call. Hence, the proposed controller constructs no internal model of the world and does not explicitly exploit changes in echo characteristics across calls. Assuming otherwise would require us to specify a segmentation and grouping mechanism by which individual echoes from subsequent calls are assigned to so-called echo-streams corresponding one-to-one with particular objects. The use of such echo-streams has been hypothesized [46] as a means for a bat’s perceptual system to organize acoustic information from complex environments. However, no explicit computational mechanism capable of the required segmentation and grouping of complex echo signals has been put forward so far. Also, while neurophysiological evidence [47, 48] for an echo stream based representation for single reflector stimuli has been found, no multiple reflector stimuli have been experimented with yet. Hence, until the possible use of an echo stream based representation in obstacle avoidance behaviour is further clarified we propose our reactive controller as a simpler and computationally explicit hypothesis. The main advantage of a reactive approach is that it considers the world as its own best model [49, 50] which is always exactly up to date and always contains every detail there is to be known [51]. By avoiding the delay due to the reconstruction of a 3D model of the environment and/or planning a path, a reactive approach results in a highly responsive and robust controller [50]. However, it should be noted that relying only on the echoes from the last call to determine the controller’s response does not make the proposed sensorimotor system memoryless. Indeed, the dynamics of the interaction between the controller and its environment introduce an implicit memory of information extracted from previous call-echo pairs. Put differently, the state of the controller, i.e. position and velocity, and latest call-echo pair jointly determine the bat’s next move, thereby ensuring that the perceptual history, i.e. previous call-echo pairs, and not just the last call-echo pair determine the controller’s response.

In the simulations, we assume the speed of the synthetic bat *v*_*bat*_ to be a function of the time of flight of the first echo, i.e. the distance to the nearest object. The range of speeds goes from 6 ms^−1^ to 0.3 ms^−1^ (see above). In addition to the speed, the flight direction also needs to be updated based on the echoes from each call. For an obstacle avoidance algorithm based on sonar, desirable flight directions are characterized by low amplitude echoes. Indeed, for the same reflector strength, weaker echoes imply obstacles that are further away or located more to the periphery. A heuristic leading to weaker echoes is to turn towards the direction of the ear which receives the weakest echoes, e.g. turning right if the right ear receives the weakest echoes.

With stationary ears, moving in the direction of the ear receiving the weakest echo would only allow for updating the horizontal flight direction. However, the ear movements of Rhinolophidae result in the main sensitivity axis of each ear to alternately point up and down. The available evidence suggests that Rhinolophidae move one ear up and the other ear down while receiving echoes [11, 12, 43]. The ears move in the other direction while receiving the next echo. In this paper, we simplified the continuous movement of the pinnae by modeling only the two extreme positions of the ears (see below and Fig 2). Considering the extreme positions of each ear results in the sonar system sampling four directions during each pair of successive calls. Therefore, we propose our controller to turn left or right depending on which ear receives the weakest echo. In addition, the controller steers up or down depending on whether the ear receiving the weakest echo is currently pointing up or down.

Echoes arriving earlier are reflected by more proximate obstacles. Hence, the initial part of the echo signal is of greater importance to an obstacle avoidance sensorimotor system. Therefore, we chose to take only the first millisecond of the echo into account (i.e. the controller only uses the onset of the echo train). We do not claim that the remainder of the echo has no function in obstacle avoidance, but we propose, as indicated by the results, that the onset of the echoes already contains sufficient information. Rhinolophidae have their ears at extreme positions in between calls and move them into the opposing configuration while receiving echoes [12]. Hence, by focussing on the onset of the echoes, we can further simplify the model and use only the extreme ear positions for each call. Apart from the resulting simplifications to our model we argue that focussing on the onset of the echoes has advantages for bats as well. Any mechanism that makes use of specific characteristics of the modulation pattern of the echo introduced by the complete pinna movement instead (e.g. [13, 14]), needs to control and/or to measure the ear movement in greater detail requiring a more complex and less robust system.

In our simulations, the echoes received at each ear *t* during the first millisecond after the arrival of the first echo are summed with randomized phase shifts. The intensity *g*_*t*_, in decibels, of the summed echoes *i* received at ear *t* is given by,

$$\begin{array}{c} g_{t}=20\cdot \log\left(|\sum_{i}10^{\frac{g_{i}}{20}}e^{j\cdot \varphi_{i,t}}|\right) \end{array}$$ (3)

In Eq (3), *ϕ*_*i*,*t*_ is a random phase angle (between −*π* and *π*) modeling the interference between narrowband echoes. Note that this phase angle is randomized independently for each reflector *i* and ear *t*. The hearing threshold was assumed to be 0 dB_*spl*_. Therefore, echo amplitudes *g*_*i*_ lower than 0 dB_*spl*_ were set to 0 and did not contribute to intensity *g*_*t*_.

We propose the bat rotates in the direction of the ear receiving the weakest echoes, given by *g*_*t*_ (Fig 3, box 5). If *g*_*l*_ < *g*_*r*_, the bat turns left. Conversely, if the right ear receives the weakest echoes (*g*_*l*_ > *g*_*r*_), the bat turns to the right. Moreover, if *g*_*l*_ < *g*_*r*_ and the left ear is pointing up (down) the bat turns up (down).

In addition to the direction of the turn, the controller also needs to specify the magnitude of the turn (Fig 3, box 6). In the proposed controller, the magnitude of the turn depends on the flight speed (which in turn depends on the distance to the closest obstacle, Fig 3, box 7). Jones and Rayner [6] report on the speed and angular rotation of *Myotis daubentonii* (See Fig 1c). We fitted a linear function to this data to obtain the following expression for angular rotation *R* in degrees per second as a function of flight speed, *R* = 665 − 116 × *V*_*bat*_. Values of *R* smaller than zero were set to zero resulting in the curve depicted in Fig 1c. Incidentally, the turning rates thus obtained correspond largely to those reported by Holderied [38].

Note that for low flight speeds the turning rate could be greatly increased. For example, Aldridge [4] reports that *R ferrumequinum* is capable of turning with a curvature of up to 115 m^−1^ (turning radius < 1 cm, angular rotation speed ∼ 1900 deg/s) when suddenly faced with a barrier. Nevertheless, as we did not aim at modeling such last minute avoidance manoeuvres, we opted for fixing the maximum turning rate to the conservative value of 665 degrees per second at *V*_*bat*_ = 0.

**Algorithm 1:** Summary of the equations governing the default controller (i.e boxes 5–7 in Fig 3). Line 2: The speed of the bat *V*_*bat*_ is set as a function *F* of the distance to the nearest obstacle *d*_*min*_ (using the curve depicted in Fig 1a). Next (lines 3–14), the speed *V*_*bat*_ is used to set the rotations of the bat in azimuth (Δ*ϕ*) and elevation (Δ*θ*). The sign of the azimuth rotation depends on the relative strength of the echoes at the left (*g*_*l*_) and the right ear (*g*_*r*_) as given by Eq (3). The sign of the elevation rotation depends on whether the ear with the weakest echo is pointing up or down.

1:	**procedure** Set Δ*ϕ*, Δ*θ*(*d*_*min*_, *g*_*l*_, *g*_*r*_)	
2:	*V*_*bat*_ ← *F*(*d*_*min*_)	▷ See Fig 1a
3:	**if** *g*_*l*_ < *g*_*r*_ **then**	
4:	Δ*ϕ* = −(665 − 116 × *V*_*bat*_)	▷ See Fig 1c
5:	**if** left ear points up **then**	
6:	Δ*θ* = +(665 − 116 × *V*_*bat*_)	▷ See Fig 1c
7:	**else**	
8:	Δ*θ* = −(665 − 116 × *V*_*bat*_)	▷ See Fig 1c
9:	**if** *g*_*l*_ > *g*_*r*_ **then**	
10:	Δ*ϕ* = +(665 − 116 × *V*_*bat*_)	▷ See Fig 1c
11:	**if** right ear points up **then**	
12:	Δ*θ* = +(665 − 116 × *V*_*bat*_)	▷ See Fig 1c
13:	**else**	
14:	Δ*θ* = −(665 − 116 × *V*_*bat*_)	▷ See Fig 1c
15:	**if** (665 − 116 × *V* *_bat_*) < 0 **then**	
16:	Δ*ϕ* = 0	▷ See Fig 1c
17:	Δ*θ* = 0	▷ See Fig 1c

In summary, the controller turns left or right depending on whether the left or the right ear received the loudest echoes. In addition, it turns up or down depending whether the ear receiving the loudest echoes is currently pointed up or down. The speed of the bat is determined by the closest (detected) obstacle (Fig 1a). In turn, the rotation speed is determined by the speed of the bat (Fig 1c). See Algorithm 1 for a listing of the computations and Fig 3 for a graphical depiction of the complete controller.

We give the synthetic bat the same aerodynamic freedom in the horizontal (left and right) and vertical plane (up and down). This is; it can turn at the same rate without taking gravity into account (but see below for a version of the controller taking into account the gravity vector). Indeed, if the synthetic bat turns upwards/downwards for long enough, it might eventually fly upside down with respect to its initial orientation. There are two reasons for modeling the vertical rotation in this way. First, while it is well known that bats are very agile, to the best of our knowledge very little information is available about the aerodynamic constraints on climbing and ascending flight of the bat. Second, and more importantly, by introducing the same constraints on both horizontal and vertical rotations, we can compare the sensorial performance of the algorithm in both the horizontal and vertical plane in the absence of differences in motor constraints. Nevertheless, we are aware that having the same constraints for both turning rates is artificial. Hence, we also test a variant of the controller that introduces a constraint on the maximum vertical rotation (see ‘constrained’ controller below).

Both *R. rouxii* and *R. ferrumequinum* emit a pulse every 80 to 90 ms on average [5, 52]. For computational ease and to simulate a lower bound update rate, the synthetic bat was simulated to emit a pulse every 100 ms. On approaching a landing site, the pulse rate of *R. ferrumequinum* was found to increase to about 80 Hz (i.e. about 12 ms interval) [9, 53]. However, the informational update of 80 Hz might not translate into an ability of the bat to update its direction 80 times a second. Rhinolophidae flap their wings at about 12 Hz (i.e. about 80 ms interval) irrespective of their air speed [54]. Considering a wing beat as the minimal unit that allows changing the direction of the flight, would allow for an update rate of at most 12 Hz. In the proposed controller, each pulse corresponds to a single update in the flight direction. Hence, as 12 Hz is very close to the modeled pulse rate of 10 Hz, the interpulse interval was fixed at 100 ms.

In the simulations, we account not only for the time needed for the echoes to arrive but also for the time required by a bat to process the echoes and produce a motor response. Übernickel et al. [55] found a reaction time of about 50 ms to transient targets in the trawling bat *Noctilio leporinus* in accordance with a similar range of reaction times 47–63 ms found in ref. [56]. Hence, we allowed for 50 ms to process the echoes. In the interval between the emission of the call and the start of the turn, the current direction and speed of flight is maintained. The interval between call and start of turn is given by (1) the time for the first echo to arrive, (2) 1 ms over which the echoes are summed and (3) 50 ms of processing time. Note that as the duration of the turn is given by the fixed call period (100 ms) minus the interval between call and start of turn, both the rotation speed *R* and the duration of the bat’s turn depend on the distance to the closest object. As the time for the first echo to arrive gets shorter, the turn duration gets longer. This increases the rotational gain of the controller even more for nearby obstacles.

### Experimental conditions

The controller was tested in 2D and 3D environments. The performance of the controller described above, referred to as the default controller from now on, was compared to that of five related controllers:

1. Fixed ears: This controller models a bat with static ears. A single directional sensitivity is used for each ear. As such, the directional sensitivity does not change from call to call. The directionality used is depicted in the top row of Fig 2b. The azimuthal rotation is updated as in the default controller. However, the sign (up/down) of the elevation rotation was selected at random.
2. Off axis pinnae: This controller is identical to the default controller. However, this controller consistently has the left ear pointing downwards and the right ear pointing upwards. If the left (right) ear receives the weakest echoes, the bat turns downwards (upwards). The azimuthal rotation is updated as before.
3. Random A: The controller differs from the default controller by randomly turning left or right at each call. While the direction of rotation is chosen randomly, the magnitude is calculated as in the default controller.
4. Random B: The same controller as the Random A variant with the addition that the rotation speed of the bat is also chosen randomly from the interval 0 to 350 degrees per second.
5. Constrained: This controller is identical to the default controller, but it constrains the angle of the bat in the vertical plane. The bat can maximally attain a climbing or descending angle of ±60 degrees.

The controller with fixed ears allows us to test the contribution of ear movement to obstacle avoidance. The controller with the pinnae fixated in an off-axis position allows us to test whether the cues necessary for obstacle avoidance are still present if the ears are fixed but are not aligned with the horizontal plane. Random A and Random B are included in the tests as baseline conditions against which to compare the other controllers. Similarly, in behavioural obstacle avoidance experiments, the performance of the bat is typically compared to the number of collisions expected from following a random path through space, e.g. [3, 25, 26]. Finally, the constrained controller adds more realistic constraints to the vertical rotation of the bats.

## Results

### Regularly spaced artificial environments

**Fig 4:**
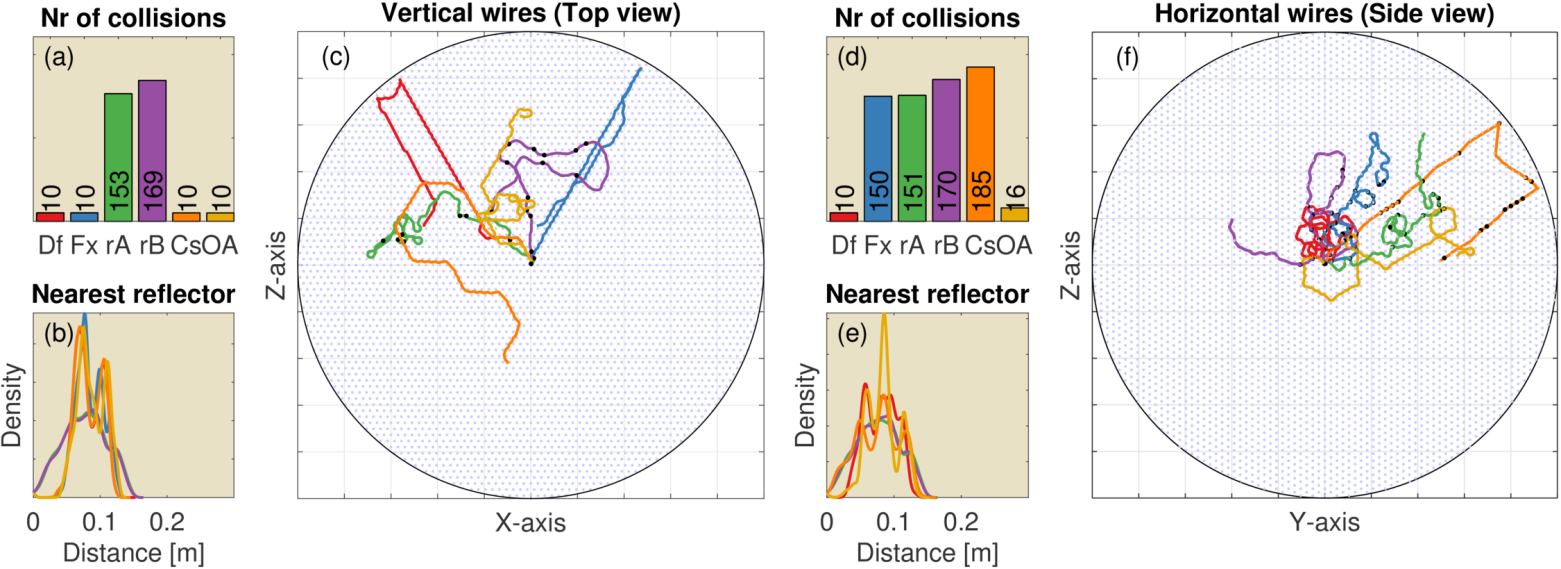
The results of 100 replications (each consisting of 250 simulated calls) of the 2D simulations in environments with regularly spaced reflectors inspired by the wire avoidance experiments of Mogdans et al. [3]. The reflectors are organized on a hexagonal grid and spaced 15 cm apart. (Left, a-c) Vertical wires. (Right, d-f) Horizontal wires (i.e. horizontal reflectors). (a) The median number of collisions for the default controller and the five variants (Df: Default controller; Fx: Fixed ears; rA: Random A; rB: Random B; Cs: Constrained; OA: Ears of axis). (b) The distribution of the distance to the nearest obstacle for each of the controllers. Colours of the lines correspond to the colours in panel (a). (c) A single example of the paths taken by each of the five controllers. The light blue dots represent the reflectors. Black dots in panel (c) indicate locations where collisions occurred. (d-f) Similar, but for horizontal wires. (f) Side view of the simulation. All simulations are started in the centre of the arena. The grid squares are 1m by 1m.

We tested the controller and its four variants in environments populated with reflectors on hexagonal grids spaced 15 cm apart (See Fig 4c and 4f for examples). In these environments, collisions are counted as the number of time steps (calls) the controller was closer than 2.5 cm to any obstacle. Hence, we modeled the synthetic bat as having a body width of 5 cm, in agreement with Mogdans et al. [3]. In most results reported below, the various controller variants differed in the resulting average distance kept from reflectors and, therefore, in their average speed and distance travelled. To compensate for this, we normalized the number of collisions for all controllers to the number of collisions per 100 m travelled.

The results show that the default controller successfully avoided both the vertical wires and the horizontal wires (Fig 4). Indeed, in these 2D tasks the number of registered collisions was much lower than in both random A and B. The controller with the fixed ears performed equally well in avoiding the vertical wires. However, avoidance of the horizontal wires was reduced to chance level by fixing the ears in the horizontal plane. In contrast, fixating the pinnae off axis, restored the obstacle avoidance performance for the horizontal wires (and did not reduce performance for the vertical wires).

The controller that was constrained in its vertical rotation performed much worse than the default controller in avoiding horizontal wires (Fig 4d). This indicates that while the moving ears supplied the necessary information to avoid obstacles, the imposed aerodynamic constraint is too restrictive to allow for successful obstacle avoidance in our grid of simulated wires.

Overall the performance results in the regularly spaced grids match the finding of Mogdans et al. [3] that obstructing the pinnae movements only interferes with the avoidance of horizontal wires, i.e. only obstacle avoidance in the vertical dimension is affected. Fixating the ears did not have an effect on the avoidance of the vertical wires. In addition, our simulations suggest that pinnae fixated in an off-axis position provide sufficient cues for obstacle avoidance in both azimuth and elevation.

### Heterogeneous artificial environments

**Fig 5:**
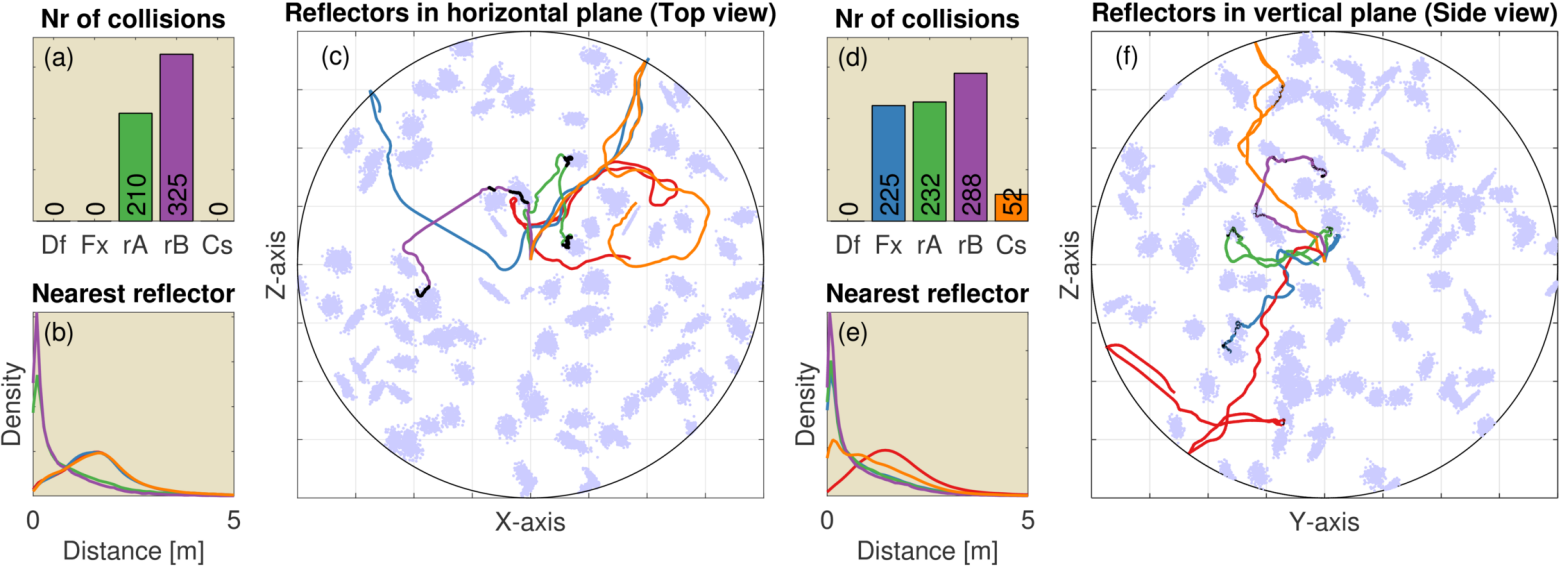
The results of 100 replications (each consisting of 250 steps) of the 2D simulation using heterogeneously spaced reflectors. Movies illustrating the behaviour of the controllers in these environments are provided as supplementary material (S1 and S2 Figs). (Left, a-c) Reflectors scattered in the horizontal plane (i.e. vertical reflectors). (Right, d-f) Reflectors scattered in the vertical plane (i.e. horizontal reflectors). (a) The median number of collisions for the default controller and the four variants (Df: Default controller; Fx: Fixed ears; rA: Random A; rB: Random B; Cs: Constrained). (b) The distribution of the distance (in m.) to the nearest obstacle for each of the controllers. Colours of the lines correspond to the colours in panel (a). (c) An example of the paths taken by each of the five controllers in a single environment. The light blue dots represent the reflectors. Black dots in panel (c) indicate locations where collisions occurred. (d-f) Similar, but for reflectors scattered in the vertical plane. (f) Side view of the simulation. All simulations are started in the centre of the arena. The grid squares are 5m by 5m.

Fig 5a and 5d show the number of collisions registered for the bat in 100 replications with reflectors scattered in either the horizontal or the vertical plane for the four variants of the controller. In these runs, collisions are defined as the number of time steps (per 100 m travelled) the controller was closer than 15 cm to the nearest obstacle, i.e. approximately half the wingspan of *R. rouxii*. The results indicate that the default controller is capable of avoiding obstacles in both the vertical and the horizontal plane. Fixing the pinnae has no effect on obstacle avoidance in the horizontal dimension. However, obstacle avoidance in the vertical dimension is reduced to chance level (i.e. similar number of collisions than controller Random A).

In the horizontal plane, the constrained controller has the same degrees of freedom as the default controller and, therefore, has the same performance. Constraining the elevation angle of the bat clearly limits its freedom. Hence, the number of collisions does increase compared to the horizontal plane. However, the number of collisions is still less than in both random baselines. The reduction in performance for the constrained controller is less dramatic than for the regularly spaced obstacles discussed above as can be seen from comparing the performance of the constrained controller in Figs 4d and 5d. We provide two movies illustrating the behaviour of the controllers in the 2D environments of Fig 5 as supplementary material.

**Fig 6:**
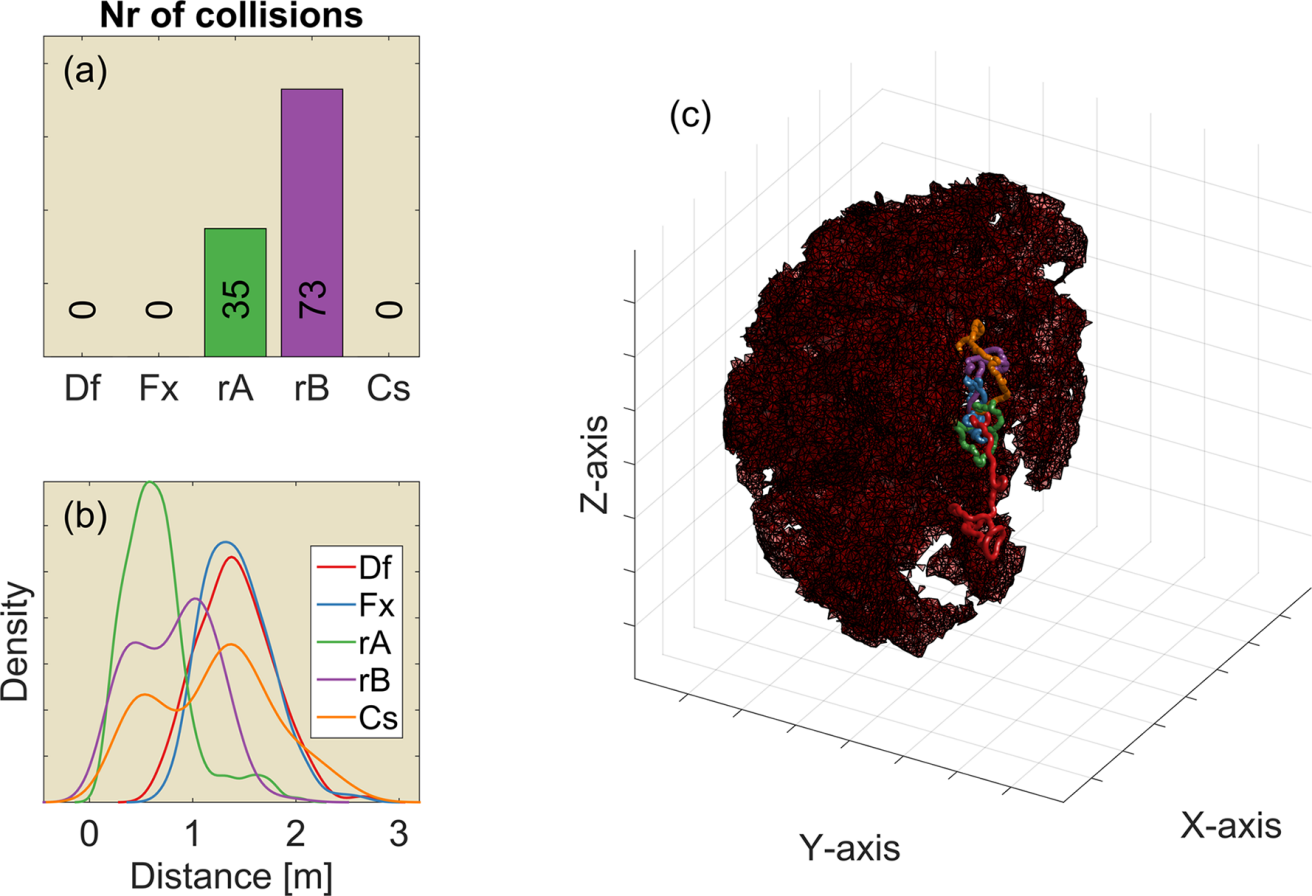
The results for 100 replications of the 3D simulated environments. This figure is also provided as a MATLAB figure in the supplementary material (S3 Fig). (a) The median number of collisions for each controller (Df: Default controller; Fx: Fixed ears; rA: Random A; rB: Random B; Cs: Constrained). (b) The distribution of the distance to the nearest obstacle. (c) Rendering of one replication of the 3D environment with the flight paths imposed. The right half of the environment has been cut away to reveal the flight paths. The grid size is 5m.

The default controller, as well as the four derived controllers, were also tested for obstacle avoidance in 3D point clouds (Fig 6, also provided as MATLAB figure in the supplementary material (S3 Fig). The default algorithm performs best. Fixing the ears does not result in an increase in the number of collisions. However, it results in flying somewhat closer to obstacles. The number of collisions does not increase by fixing the ears as the controller is still able to avoid obstacles in the horizontal plane. This implies the controller with the fixed ears solves the 3D obstacle avoidance problem as a sequence of 2D problems. Indeed, the 3D point clouds do not require the controller to perform obstacle avoidance in both horizontal and vertical plane simultaneously, it can avoid collisions by avoiding obstacles in a single plane.

The two random controllers performed worse than the default controller with a drastic increase in the number of collisions. The constrained controller performed at the same level as the default controller with respect to the number of collisions. Hence, the reduced freedom in elevation rotation does not seem to hamper this controller in this environment.

### Tilted torus environment

**Fig 7:**
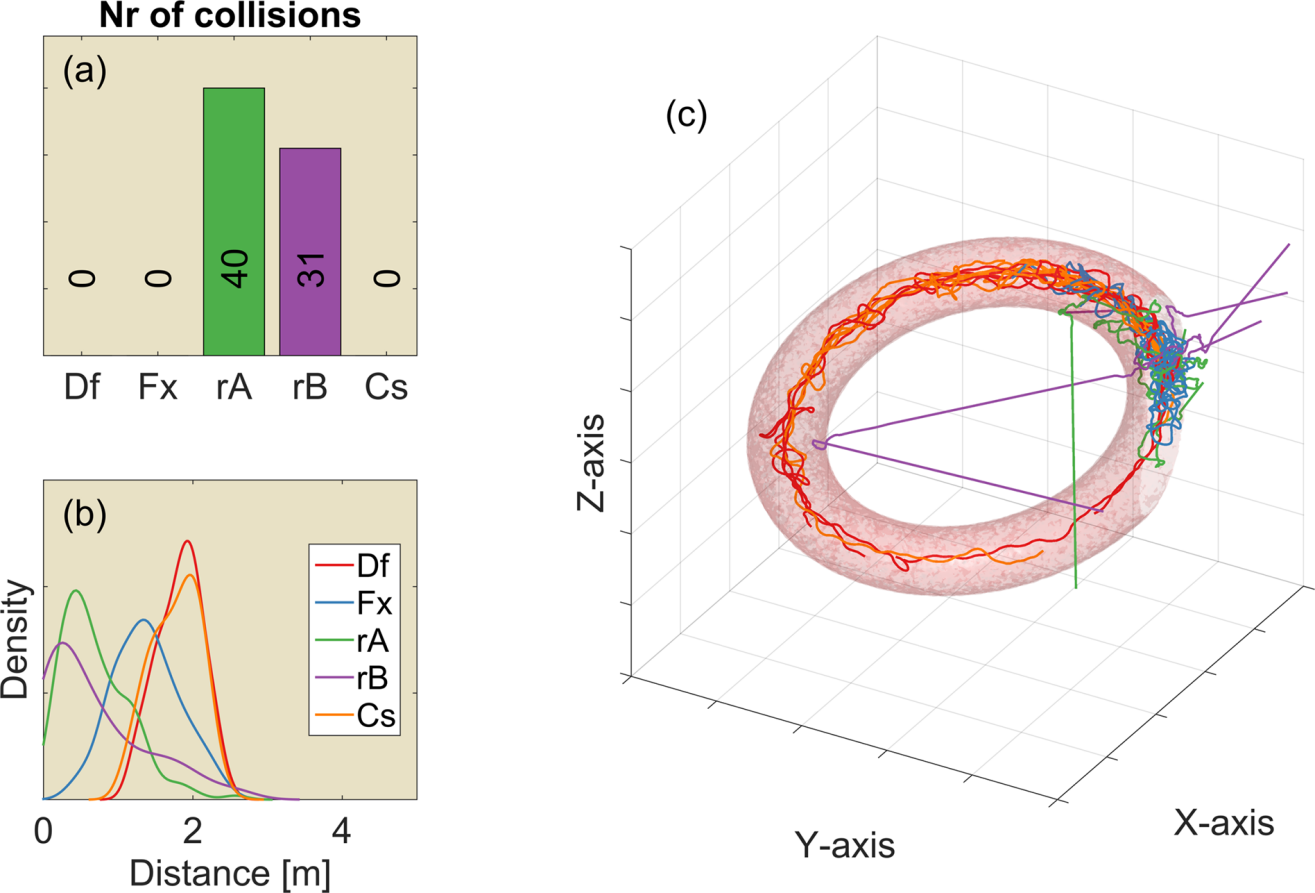
Results of 25 replications in the tilted torus environment (each replication consists of 250 steps). This figure is also provided as a MATLAB figure in the supplementary material (S4 Fig) (a) The median number of collision per replication corrected for distance travelled (b) Distribution of the distance to the nearest obstacle for each of the controllers (Df: Default controller; Fx: Fixed ears; rA: Random A; rB: Random B; Cs: Constrained). (c) Plots of the paths for 5 replications in the torus environment (not all replication were plotted for reasons of clarity). The torus is rendered as a transparent volume. The individual reflectors making up the torus are not plotted as they would obscure the flight paths. The grid size is 5m.

The tilted torus environment explicitly tests whether the controller(s) can follow a corridor in both azimuth and elevation. The results depicted in Fig 7 show that the random controllers result in more collisions (Fig 7a) and flying closer to reflectors (Fig 7b, (also supplied as a MATLAB figure in the supplementary material (S4 Fig) than the other controllers. The number of collisions follows a similar pattern as the number of collisions in the 3D environment depicted in Fig 6. However, more importantly, only the controllers with moving ears (i.e. the Default and Constrained controllers) succeed in following the torus. The random controllers often exit the torus quickly, explaining the low number of collisions for the controller Random B. The controller with fixed ears stays in the torus without colliding but is unable to complete a circular path inside the torus. It is confined to a subsection of the torus.

### Laser scanned environments

**Fig 8:**
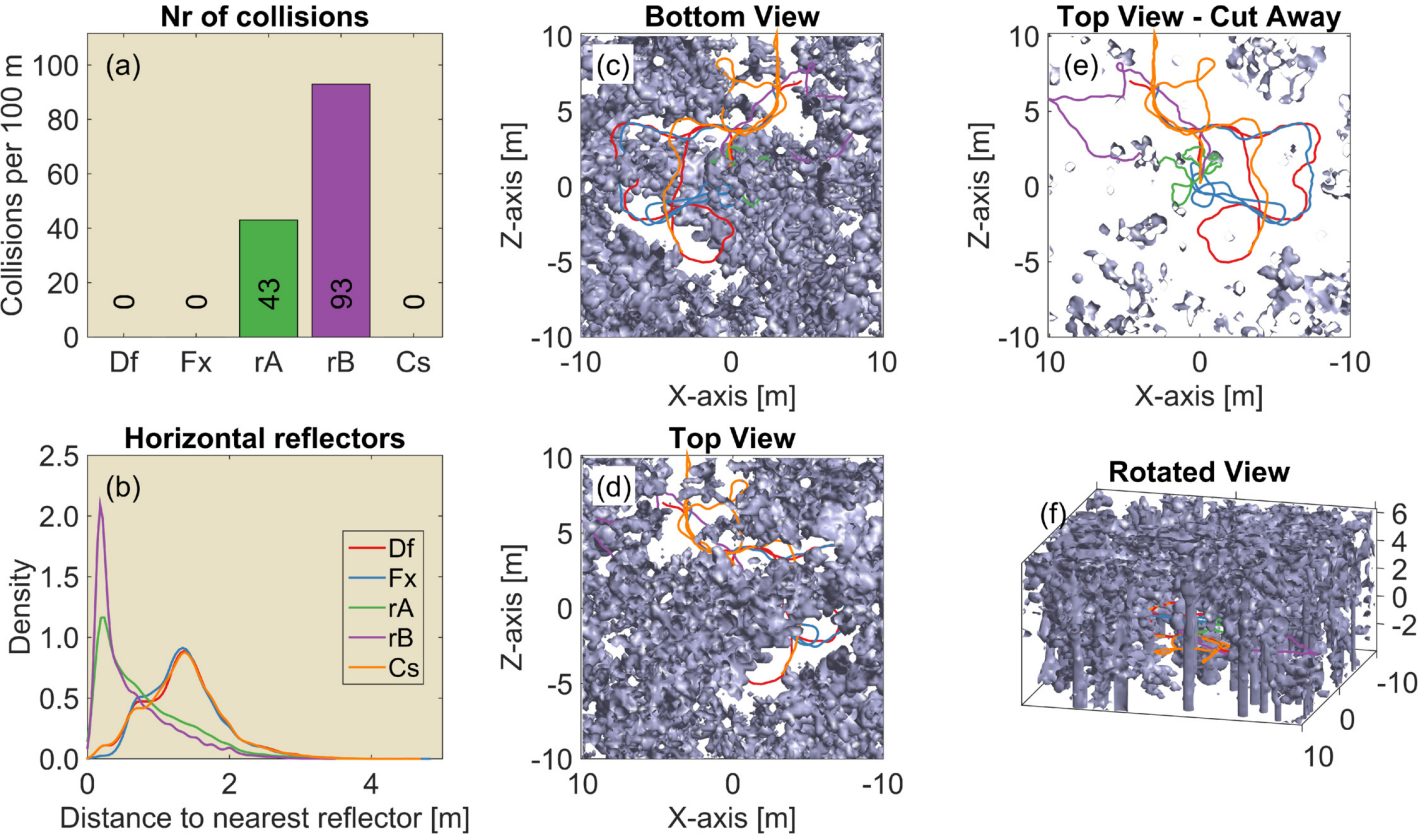
Results for the 3D scanned forest patch. (a) The median number of collisions for 50 replications of the experiment (Df: Default controller; Fx: Fixed ears; rA: Random A; rB: Random B; Cs: Constrained). (b) The distribution of the distance to the nearest obstacle. (c-f) Renderings of the obstacles with a single example flight path overlaid. Colours indicate the different controllers. Each run consisted of 250 steps. The 3D rendering is also provided as a MATLAB figure in the supplementary material (S5 Fig).

**Fig 9:**
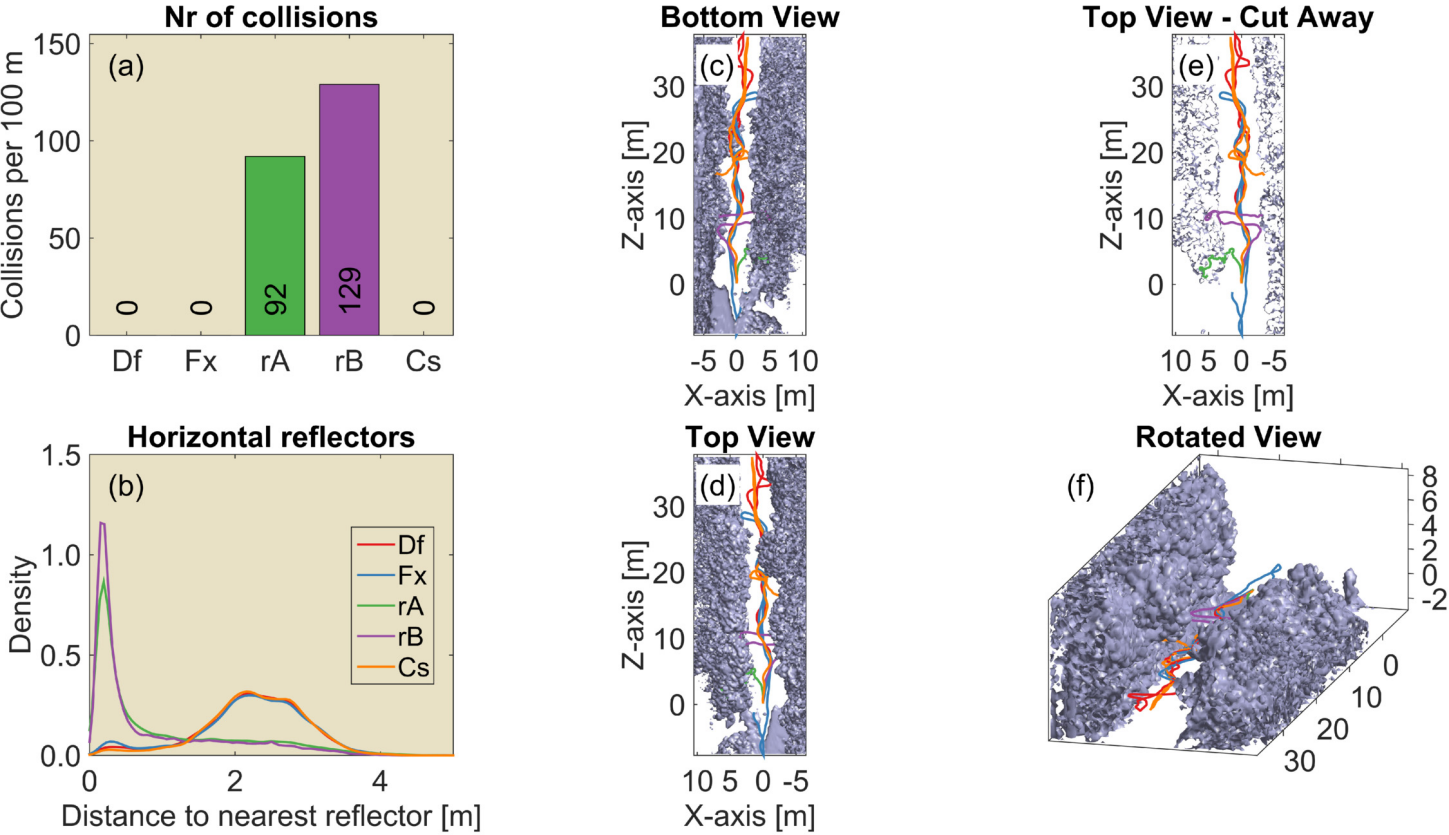
Results for the 3D scanned forest road. (a) The median number of collisions for 50 replications of the experiment (Df: Default controller; Fx: Fixed ears; rA: Random A; rB: Random B; Cs: Constrained). (b) The distribution of the distance to the nearest obstacle. (c-f) Renderings of the obstacles with a single example flight path overlaid. Colours indicate the different controllers. Each run consisted of 250 steps. The 3D rendering is also provided as a MATLAB figure in the supplementary material (S6 Fig).

Fig 8 shows the results of 50 replications of the experiment using the 3D scanning data from the fir forest. Likewise Fig 9 shows the results of 50 experimental runs using the 3D scan of the forest corridor. As real bats show nearly 2D flight behaviour in similar real environments (as found e.g. in Holderied [57]), we ignored the elevation commands of the controller resulting in 2D flight paths in these simulations. In both environments, Random A and B performed substantially worse than any other variant. Note that, in these experiments, while the bat’s flight path is restricted to a plane the echo signals the controller derives its decisions from are calculated based on the full 3D environment.

## Discussion

Echolocation supports the execution of many tasks varying widely in computational complexity such as object recognition [16, 58], prey localization [22, 59], finding water [60] and navigation [61]. Understanding the echolocation ability of bats can be thought of as isolating the (minimal set of) cues needed to perform each of these different tasks and confirming the sufficiency of those cues in behavioural experiments [36, 60], simulations [13, 14, 20, 62] or robotic studies [15, 20, 63, 64]. For some tasks, a minimal set of sufficient cues has been determined. For example, water bodies can be identified as horizontal reflective surfaces. Indeed, any horizontal surface with the correct reflective properties is readily mistaken by bats as a water surface [60]. Other tasks for which a minimal set of cues has been determined include the recognition of flower size [36] or prey size [65, 66]. However, while the ability of bats to avoid obstacles was the first to be studied (e.g. [26] and reviewed in [25]) following the groundbreaking experiments by Lazzaro Spallanzani (1729-1799, described in [67]), relatively little is known about the minimal set of cues sufficient to support obstacle avoidance, one of the most basic echolocation supported tasks [68].

One explanation for this hiatus seems to be the assumption that the bat’s ability to avoid complex obstacles relies on the more basic competence of reconstructing the 3D layout of its surroundings first. It has been suggested [3, 69] and verified [15] that Rhinolophidae, using long narrowband calls, could localize single targets using the changing IID cues generated by their moving ears. However, complex reflectors in bat habitats, such as plants and trees [16], return many overlapping echoes. The multitude of echoes returned by natural obstacles is problematic for such a localization strategy because none of the proposed cues has been demonstrated to be robust in the face of many overlapping echoes. Similarly, bats using frequency modulated calls can locate single targets based on binaural spectral cues, e.g. [59, 62, 70, 71]. However, due to the temporal integration in the bat’s auditory system [72, 73], spectral cues will also degrade when faced with many overlapping echoes [74]. In addition, spectral cues are unreliable for low amplitude echoes [62]. As a growing body of research on bat echolocation shows that bats can cope with extremely challenging situations (e.g., [27, 75]), we conclude that the cues used for obstacle avoidance must be robust and available even (or, especially) in situations where a multitude of complex objects generate many overlapping echoes. Hence, we argue that it is unlikely that a 3D reconstruction capability would be a precondition for successful obstacle avoidance.

**Fig 10:**
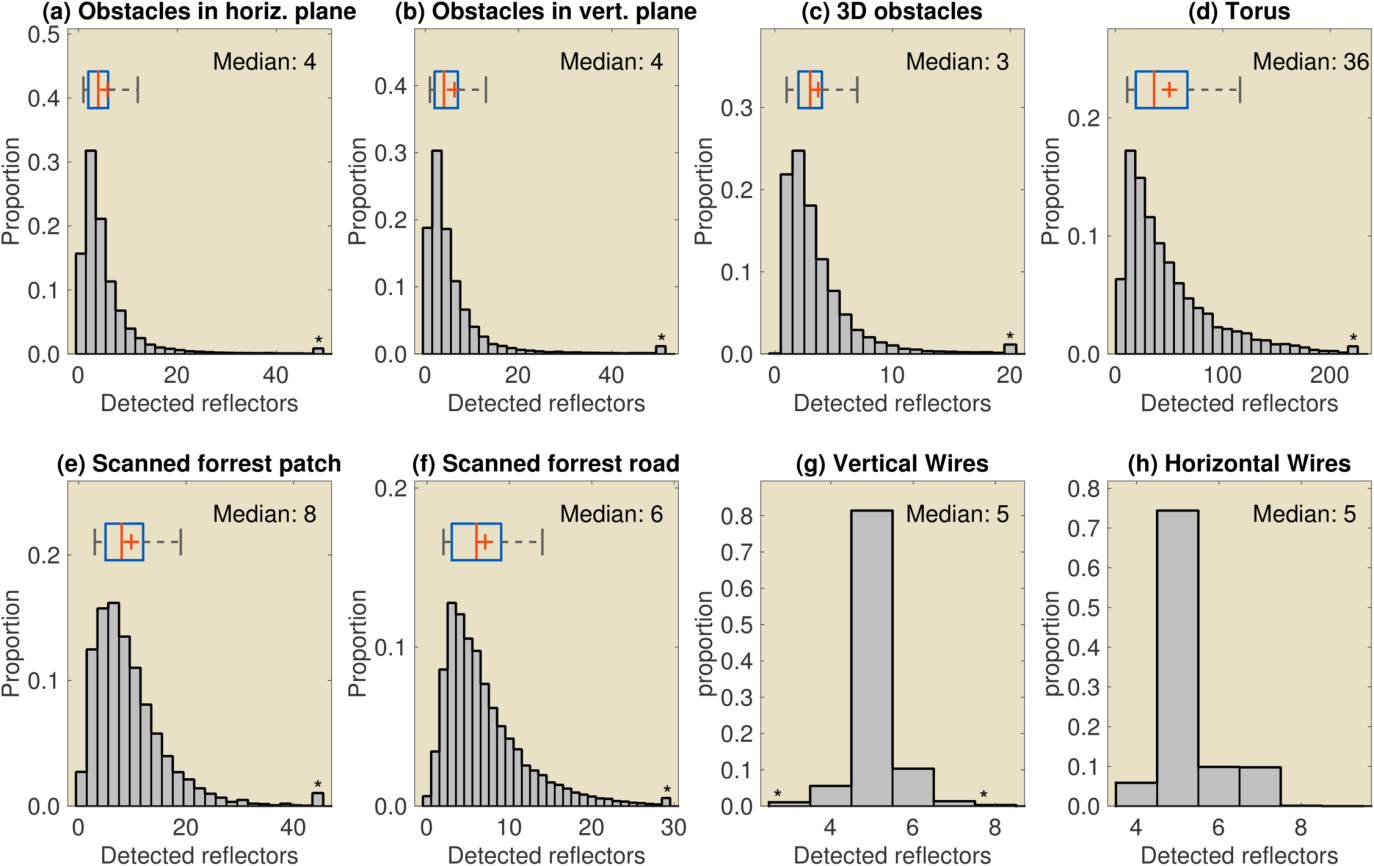
Histograms of the number of detectable echoes as received by the artificial bat within the first millisecond after the first echo, i.e. the number of echoes used by the controller to steer the bat. The data plotted is for the default controller. As the left and the right ear do not necessarily detected the same (number of) reflectors for each call, the data is the maximum number of echoes across the left and the right ear per call. (a) Data for the 2D simulation using heterogeneously spaced reflectors in the horizontal plane (Fig 5a-5c). (b) Data for the 2D simulation using heterogeneously spaced reflectors in the vertical plane (Fig 5d-5f). (c) Data for the 3D simulated environments (Fig 6). (d) Data for the tilted torus environment (Fig 7). (e-f) Data for the scanned forest patch and road respectively (Figs 8) and 9. (g) Data for the 2D simulations in environments with regularly spaced vertical wires (Fig 4a-4c). (h) Data for the 2D simulations in environments with regularly spaced horizontal wires (Fig 4d-4f). A star (*) indicates the x-axis was cropped, and the remaining data was lumped into the rightmost bin. The boxplot in panels (a-f) uses a red line for the median, a red cross to indicate the mean, a blue box around the 25% and 75% quartiles and whiskers to bounding both 9% and 91% of the data. The boxplot was omitted from (g) and (h) due to low spreading in those panels.

In this paper, we propose an alternative obstacle avoidance strategy that does not rely on explicit 3D reconstruction. This strategy is capable of obstacle avoidance, even when faced with complex obstacles returning overlapping echoes. Indeed, even though the controller only uses the first millisecond of the returning echo train, this short interval typically contained echoes from multiple reflectors (see histograms in Fig 10). The median number of reflectors returning a detectable echo within the first millisecond varied across simulations. For example, the median number of echoes returned by the 3D artificial environment was 3. In contrast, the median number of detectable echoes returned by the torus was 36. In addition to variation across conditions, the number of detectable echoes also varies from call to call within conditions. This is demonstrated by the long tails of the distributions.

Our results from both 2D and 3D simulations in artificial and natural environments show that the IID and delay cues derived from the onset of the first echo when combined with characteristic ear movements are sufficient to support obstacle avoidance using the bat *R. rouxii* as a model. It is true that, while this minimal set of cues seems sufficient to avoid obstacles in most cases, the controller did fly into obstacles on a number of occasions, e.g. Fig 4. However, real bats are not perfect at avoiding obstacles either and they sometimes have to fall back upon some last moment collision avoidance behaviours, presumably if echoes become too loud or too close [25]. For instance, Aldridge [4] found that *R. ferrumequinum* was capable of turning with an angular velocity of up to 1900 degrees per second when suddenly faced with a barrier. Therefore, bats are capable of performing very agile last minute evasive manoeuvres. Such behaviour was not programmed into the controller but could have avoided collisions in the limited number of instances where the synthetic bat ventured too close to obstacles.

An interesting further result pertaining particularly to constant frequency bats is the matching of the experimental results of Mogdans et al. [3]. In spite of their limited magnitude (about 30 degrees, see Fig 2), the success of our default controller confirms that ear movements can explain obstacle avoidance in the vertical plane (Fig 5). Furthermore, as in the experiments of Mogdans et al. [3], fixing the ears reduced the synthetic bat’s ability to avoid horizontal wires while leaving the ability to avoid vertical wires intact. Therefore, this obstacle avoidance study, by suggesting a specific mechanism, adds further evidence in favor of the functional relevance of these small ear movements for constant frequency bats.

A prediction following from our proposed controller is that cyclic ear movements are not strictly necessary for obstacle avoidance. The controller only uses a snapshot at the onset of the cycle. Indeed, for obstacle avoidance in the horizontal plane the controller relies on the different azimuthal directions in which both ears point. Similarly, for obstacle avoidance in the vertical plane, the controller only requires the ears to be pointing in different elevation directions. We tested this prediction using ears that were fixed in an off-axis position (Fig 4, controller with off-axis ears). The results confirmed the prediction. Hence, we suggest that Rhinolophidae with their ears fixed in an off-axis position have access to sufficient information to avoid obstacles in both planes. In this respect, it is interesting to note that Mogdans et al. [3] report: “Single photographic flight records of intact bats revealed that bats sometimes passed vertical wires with the head tilted off the horizontal plane”. Such head tilting would have a similar effect than fixing the ears off-axis as we did in our experiments. Please note that we do not want to imply that the cyclic ear movements do not provide additional essential information that can be used to control other behaviours, e.g. the localization of individual reflectors such as prey [13–15].

The controller proposed in this paper is dependent upon the sign of the IID only: it turns left/right and up/down based on which ear receives the lower echo amplitude. Therefore, the algorithm supposes robustness against any alterations of the head related transfer function that preserve the tendency for ipsilateral reflectors to be louder than contralateral ones. Early experiments have shown that FM bats of which both pinna and tragus were removed avoided obstacles just as well as bats with intact ears [28]. Furthermore, disrupting the IID cues by plugging one ear reduces the obstacle avoidance performance of *R. ferrumequinum*. Plugging both ears lightly (attenuation 15–25 dB) does not deteriorate obstacle avoidance [76]. Plugging both ears more tightly (echo attenuation 55–60 dB, [76]) or completely [28] reduces obstacle avoidance performance presumably by preventing echoes from being detected. These results indicate that crude binaural intensity cues, those that are unaffected by the removal or the plugging of both ears but are affected by changing the sensitivity of a single ear, are sufficient to avoid obstacles. These results are in agreement with the predictions from the proposed controller, as it relies only on the sign of the IID. In contrast, these early findings [28, 76] can not be explained by assuming that obstacle avoidance depends on a 3D reconstruction of the bat’s surroundings. Indeed, deforming the outer ears by gluing the tragus forward to the side of the head has been shown to increase sound localization errors in FM bats [59, 70, 77]. A 3D reconstruction would imply the bat can simultaneously localize multiple reflectors in both azimuth and elevation. This seems to require the presence of intact pinnae [59, 70, 77], which was not the case in the study of Hahn [28]. Since he found that the bats could still avoid obstacles without pinnae a 3D reconstruction of the obstacle layout does not seem necessary for obstacle avoidance.

It should be noted that the experiments reported in ref. [28] were not conducted in the dark. Therefore, bats might have relied both on vision and on echolocation. Nevertheless, depriving the bats of their hearing by filling the meatus with plaster (but not the removal of the tragi and pinnae) resulted in increased collisions. Hence, while the bats could be relying partly on vision in ref. [28], they were clearly echolocating as well and could not solve the obstacle avoidance task by relying on vision alone.

**Fig 11:**
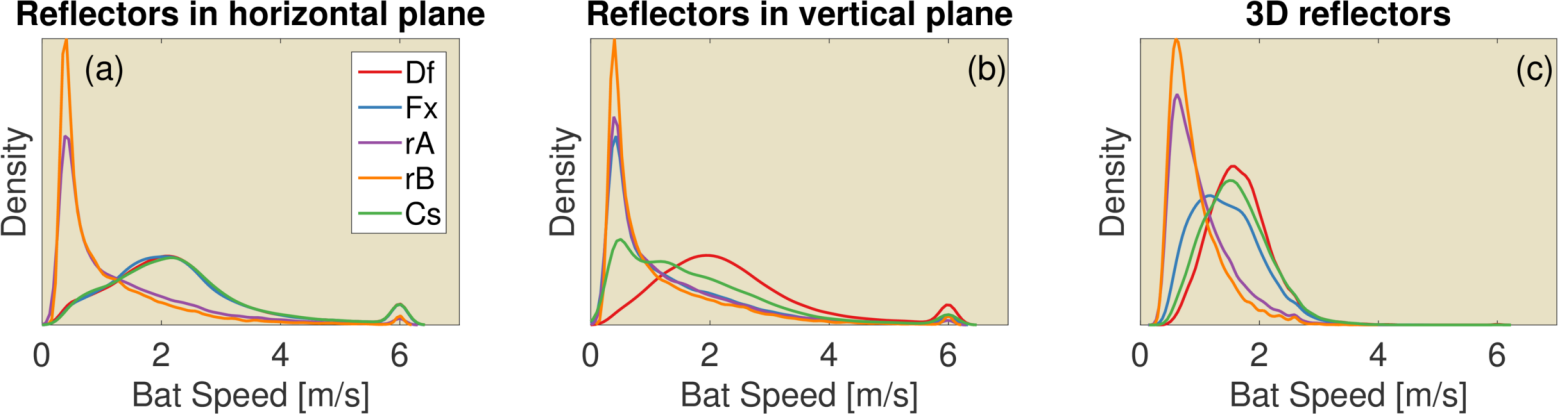
The distribution of the flight speeds in three different simulated environments. (a) Reflectors in the horizontal plane (see Fig 5a-5c). (b) Reflectors in the vertical plane (see Fig 5d-5f). (c) 3D reflectors (see Fig 6)

To validate the plausibility of the flight speeds generated by the proposed controller we evaluated the simulated flight speeds for a number of the simulations discussed above by plotting their distribution (Fig 11). From these plots, we conclude that the flight speeds are realistic. The default algorithm resulted in an average speed of about 2.2–2.3 ms^−1^. Fawcett and Ratcliffe [78] reported on the flight speed of untrained *M. daubentonii* in a small and a large flight room with a ground surface area of 3 m × 3 m and 7 m × 4.8 m respectively. The weight of this species ranges from 5 to 10 grams [79]. Commuting speeds between 3 and 8 ms^−1^ have been reported [80]. In the experiments of Fawcett and Ratcliffe [78], single bats adopted an average flight speed of about 2.2 ms^−1^ in the larger flight room and 1.3 in the smaller flight room. Hence, the average flight speed used by our default algorithm is very close to the flight speeds reported for the larger flight room. Hence, although we were unable to find flight speeds for *R. rouxii*, evidence from another species corroborates our simulated flight speeds.

### Generalization to FM bats

The results presented in this paper can be readily extended to bats using frequency-modulated (FM) calls. For obstacle avoidance in the horizontal plane, this extension follows directly from our results. Indeed, in our simulations the controller avoids obstacles in the horizontal plane by using first echo delay and IID extracted from a single narrow frequency band. Moreover, the bat only processes the onset of the echo (i.e. the first millisecond). This type of transient information is also available to bats using FM signals. The main difference between FM and CF bats in this respect is that FM bats have access to IIDs across multiple frequency bands. Bats navigating along hedgerows [57] or among the trunks of trees could make use of this horizontal obstacle avoidance mechanism.

To demonstrate that the proposed mechanism indeed extends to FM bats avoiding obstacles, we modeled an FM bat flying in heterogeneous artificial environments (identical to those used in Fig 5). The controller was adapted to use the head related transfer function [81] and emission directivity [82] of the FM bat *Phyllostomus discolor* at 60 kHz (atmospheric attenuation: 2 dB/m [35]). *P. discolor* uses frequency modulated calls which include frequencies between 40 and 90 kHz [83, 84]. However, in the current simulations, we simulated only one of the frequency channels available to this bat. i.e. we modelled a single frequency channel at 60 kHz. No cyclic ear movements were simulated. In addition, as FM bats do not compensate for Doppler shifts this behaviour was omitted. Apart from these changes, the controller was not altered. In flight, the calls of *P. discolor* have been reported to reach a peak intensity of 124 dB (cited in [85]). Hence, we used 120 dB as emission strength (*g*_*bat*_, Eq (1)) as before. The maximum gain of the HRTF was set to 6 dB [86].

**Fig 12:**
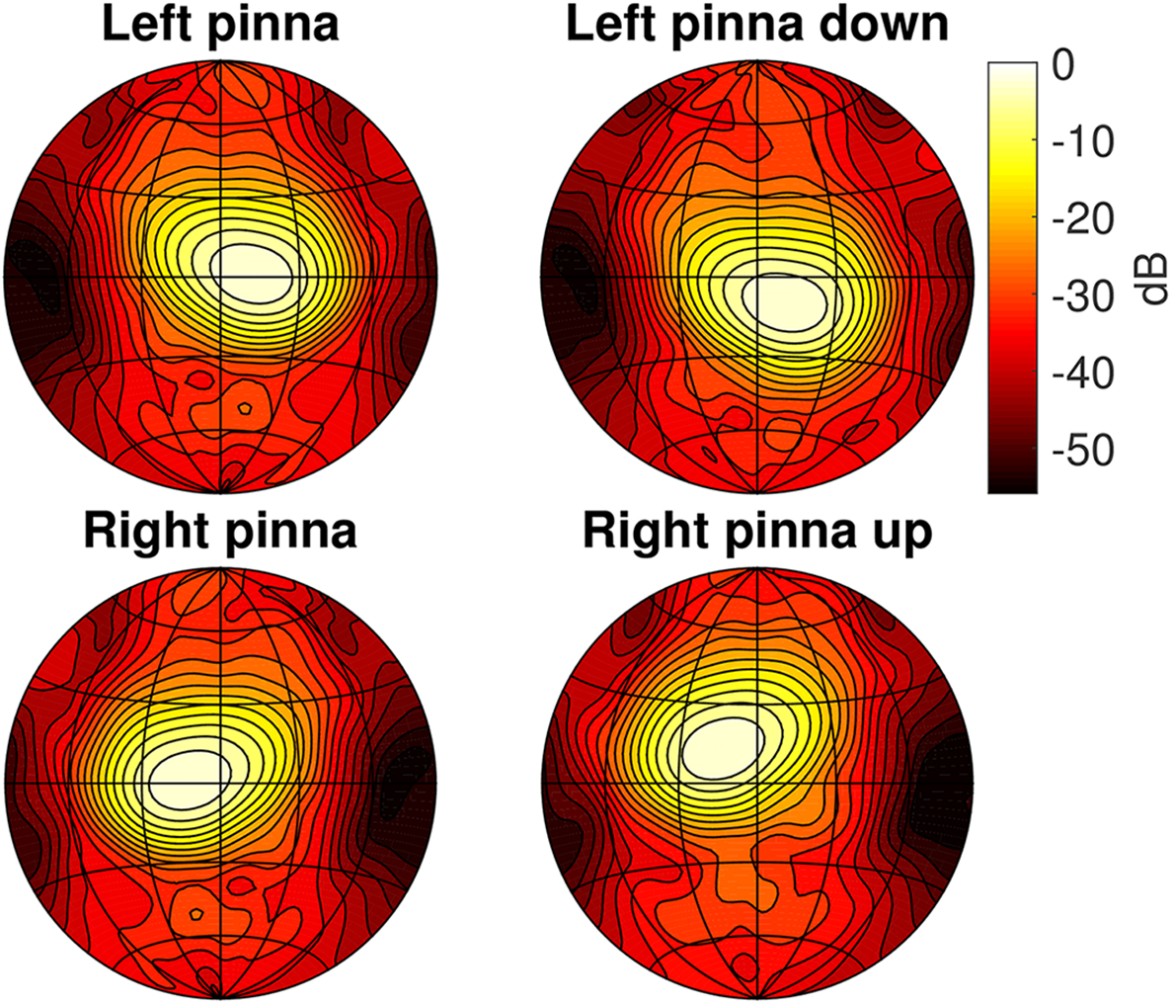
The simulated directional sensitivity at 60 kHz for *P. discolor* [81, 82] (combination of the head related transfer function (HRTF) and the emission beam directionality). Note that only the HRTF for a single (left) ear was available. Hence, the right ear HRTF was created by mirroring the left ear directionality. The directionality plots have been normalized to a maximum of 0 dB, and the contour lines are spaced 3 dB apart.

To the best of our knowledge, ear movements of FM bats in flight have only been studied in the final approach during prey capture, e.g. [22, 87]. Hence, it is unknown whether FM bats exhibit ear motions while avoiding obstacles. However, as indicated above, ear movements are not necessary for successful obstacle avoidance in the vertical plane. Indeed, the controller with pinnae fixed in an off-axis position performed nearly as well as the default controller (see Fig 4). Therefore, we hypothesize that FM bats might be able to avoid obstacles in azimuth as well as elevation by turning their pinnae off-axis. We tested this by combining the controller with an HRTF obtained by rotating the left ear down by 15 degrees and the right ear up by 15 degrees (see Fig 12). This is the same rotation of the pinnae as used for the simulations of *R. rouxii*. We used the same configuration of the simulated ears for the Constrained controller. The default controller, on the other hand, has both ears co-located in the horizontal plane (see Fig 12).

**Fig 13:**
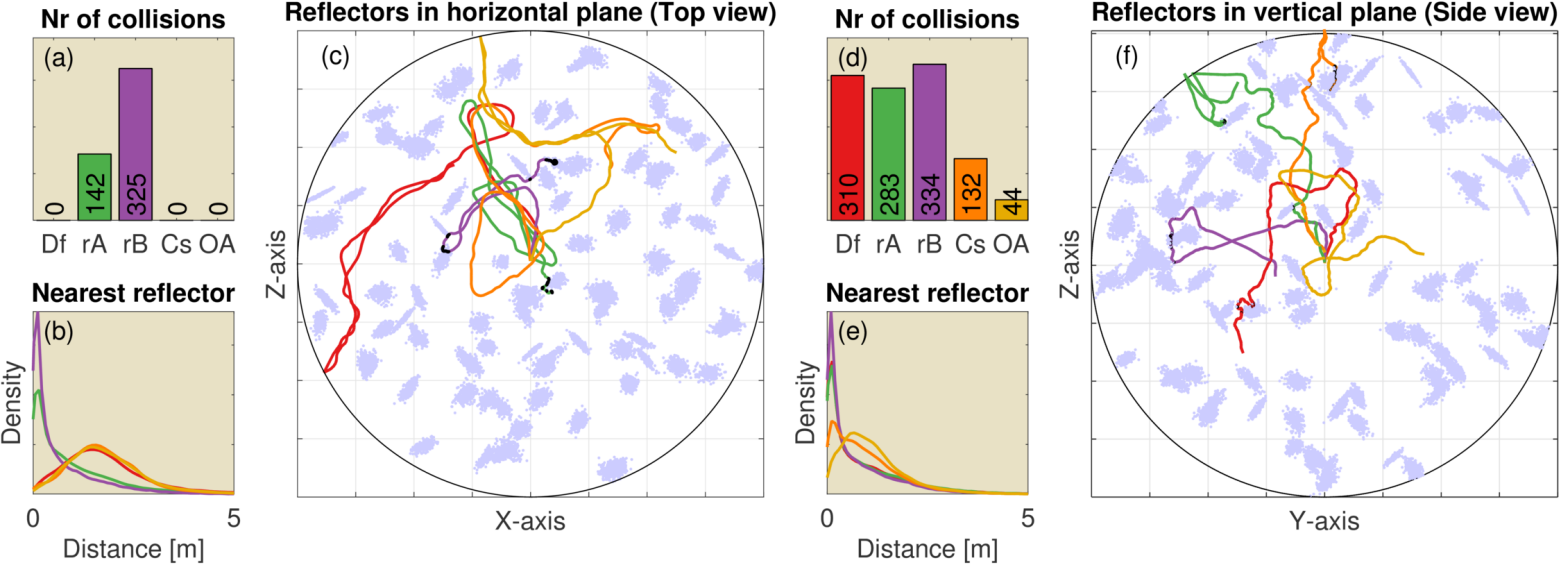
The results for 100 replications of *P. discolor* (an FM bat) flying in a heterogeneous artificial environment. (Left, a-c) Reflectors scattered in the horizontal plane (i.e. vertical reflectors). (Right, d-f) Reflectors scattered in the vertical plane (i.e. horizontal reflectors). (a) The median number of collisions for the default controller and the four variants. ‘Off-axis’ indicates the controller using an HRTF obtained by rotating the left ear downwards by 15 degrees and rotating the right ear upwards by 15 degrees (right column Fig 12). The ‘Constrained controller’ used the same ear configuration. In addition, the vertical rotation of this controller was constrained as before. All other controllers used the HRTF with the ears in the default position (left column Fig 12) (b) The distribution of the distance (in m.) to the nearest obstacle for each of the controllers. Colours of the lines correspond to the colours in panel (a). (c) An example of the paths taken by each of the five controllers in a single environment. The light blue dots represent the reflectors. (d-f) Similar, but for reflectors scattered in the vertical plane. (f) Side view of the simulation. All simulations are started in the centre of the arena. Black dots in panels (c) and (f) indicated locations were collisions occurred.

Fig 13a-13c shows that, as expected, the controller using the *P. discolor* directionality can avoid obstacles in the horizontal plane. The only controller variants that were unable to avoid obstacles were Random A and Random B. Pointing the ears off axis did not have an (adverse) effect on the obstacle avoidance behaviour. The results in Fig 13d-13f show that equipping the FM bat controller with ears pointing off-axis results in increased obstacle avoidance performance. In contrast, having the ears co-located in the horizontal plane (i.e. the Default controller) leads to numerous collisions.

Rotating their ears into an off-axis position is only one way in which FM bats could compensate for the absence of cyclic ear movements during flight. They could also change the orientation of their heads and/or bodies between calls. In fact, this behaviour has been observed in CF bats when being prevented from rotating their pinnae. Mogdans et al. [3] reported that in their experiment, the CF bats with immobilized pinnae showed more vigorous head movements than before surgery and compared to the controls while hanging in the flight room. They also reported, as referred to above, that flight records of intact bats revealed they sometimes passed vertical wires with the head tilted off the horizontal plane. Evidence for changes in head orientation in FM bats has been reported for *Eptesicus fuscus* which has been shown to be able to shift its beam from call to call, e.g. [88]. Likewise, pipistrelle bats were found to exhibit extensive scanning behaviour in azimuth and elevation while flying through natural habitats [89]. This behaviour in combination with the mechanism proposed above, i.e. rotate towards the ear receiving the weakest echo, would support obstacle avoidance based on the same cues used by our controller.

### Behaviour-based control for echolocating bats

The sensorimotor strategy proposed in this paper can be readily incorporated into a behavior-based control architecture. This type of controller, originally proposed for robots by Brooks [90] and inspired by neuroscience [50], decomposes complex behavior into a number of independent sensorimotor loops (reviewed in refs. [50, 91, 92]). Each sensorimotor loop controls a single behaviour such as obstacle avoidance, approaching targets or corridor following. All sensor data is fed into each loop. However, loops only extract the information necessary for the behaviour they control. An action selection mechanism (e.g. mutual inhibition of behaviours [90]) ensures that only a single sensorimotor loop drives the actuators [93] at each point in time.

Brooks proposed the behaviour-based control architecture as an alternative to so-called deliberate control architectures. These controllers process the sensor data to derive a general representation of the world first. Once a general and complete representation has been derived, planning and reasoning algorithms are employed next to determine the most suitable action sequence [49]. However, deriving a representation that supports all required actions has proven to be the most challenging aspect of deliberate controllers. Indeed, experience in robotics has learned this is only possible for highly simplified environments. Today, no autonomous robot operating in realistic environments is operated by an entirely deliberate control architecture [91]. In contrast, behaviour based controllers avoid having to compute explicitly an internal representation of the world. Indeed, in the words of Brooks, in a controller consisting of multiple sensorimotor loops

*..the notion of perception delivering a description of the world gets blurred […] as the part of the system doing perception is spread out over many pieces […]. Certainly there is no identifiable place where the output of perception can be found.* [90]

We argue that the fact that behaviour-based control does not depend on the extraction of a general representation of the environment makes it an appealing candidate as a control strategy in echolocating bats. Indeed, the sparseness and unreliability of localization cues makes deriving a general representation of the world very difficult, if not impossible under many real world conditions. A behaviour-based control architecture would circumvent this issue by only relying on extracting (and possibly storing [94]) those cues necessary for a particular sensorimotor loop.

Furthermore, behaviour-based control architectures readily allow for redundancy. Each behaviour (e.g. obstacle avoidance [90]) can be controlled by multiple, independent sensorimotor loops each exploiting different cues. For example, in the current paper we have proposed a sensorimotor loop for obstacle avoidance based on IID and time of flight cues derived from the onset of the first echo. However, we acknowledge that bats may use many more echo cues than the ones we have exploited in this paper. Also, they are likely to integrate more echo information across calls, i.e. base their decisions on echo-stream information. In particular, CF bats might be using the complete echo for extracting IID, use Doppler shifts or use the FM parts of the echoes. FM bats, on the other hand, are very likely to use spectral cues whenever available. Each of these cues could be extracted, evaluated, stored and mapped to motor commands by a set of dedicated sensorimotor loops taking precedence through an adequate action selection mechanism. This would lead to a high level of robustness as, in case a particular sensorimotor loop fails to extract the relevant cues, other loops will take over motor control.

In summary, we tentatively propose that many aspects of bat echolocation—including prey capture, obstacle avoidance and navigation—could be modeled by a behaviour-based control architecture consisting of a set of sensorimotor loops each extracting and exploiting a subset of cues from the echoes. Indeed, other sensorimotor loops proposed in the past fit readily in this framework, e.g. the prey capture strategies proposed by Kuc [20] and Walker et. al. [15] or the models of target approach proposed by Lee et. al. [95] and Bar et al. [96]. In the case of obstacle avoidance, we consider the proposed obstacle avoidance behaviour to be a robust sensorimotor loop to which both FM and CF bats can fall back on in case less reliable cues are unavailable. We maintain that a behaviour-based controller would result in a robust echolocator capable of exploiting a wide range of cues whilst keeping computational demands limited by avoiding the need to reconstruct a general representation of the environment from noisy and complex echoes.

Proposing a behaviour-based architecture as a model for echolocation based control in bats implies that future research should not only focus on identifying sensorimotor loops underlying different behaviours but also on how these loops interact and how context-dependent action selection is achieved. Indeed, a behaviour-based controller offers a framework in which to analyze the bats’ flexibility in exploiting a variety of (multimodal) cues under changing circumstances, e.g. [74].

### Conclusion

In conclusion, we propose that Interaural Intensity Differences calculated on the onset of the first echo, in combination with first echo delay, constitute a sufficient set of stable and robust cues for avoiding obstacles in a 3D world—without the need to reconstruct the 3D layout of the reflectors from complex and noisy echo signals. Our simulations suggest that exploiting these cues would allow both FM and CF bats to perform this basic echolocation subtask with a limited computational load and minimal latency providing a hard real-time response capability.

## Supporting Information

S1 Fig Movie illustrating the behaviour of the controllers in a 2D environment with reflectors in the horizontal plane (see Fig 5a-5c). (AVI)

S2 Fig Movie illustrating the behaviour of the controllers in a 2D environment with reflectors in the vertical plane (see Fig 5d-5f). (AVI)

S3 Fig Fig 7 in MATLAB figure format. (FIG)

S4 Fig Fig 6 in MATLAB figure format. (FIG)

S5 Fig Fig 8 in MATLAB figure format. (FIG)

S6 Fig Fig 9 in MATLAB figure format. (FIG)
